# Update on novel, validly published, and included bacterial taxa derived from human clinical specimens and taxonomic revisions published in 2025

**DOI:** 10.1128/jcm.00973-26

**Published:** 2026-09-04

**Authors:** Arianna Carella, Karen C. Carroll, Erik Munson

**Affiliations:** 1 Department of Medical Laboratory Science, Marquette University 5505, Milwaukee, Wisconsin, USA; 2 Division of Medical Microbiology, Department of Pathology, The Johns Hopkins University School of Medicine 1500, Baltimore, Maryland, USA; 3 Wisconsin Clinical Laboratory Network, Laboratory Technical Advisory Group, Madison, Wisconsin, USA; Medical College of Wisconsin, Milwaukee, Wisconsin, USA

**Keywords:** taxonomy, nomenclature, prokaryotes, human clinical specimens

## Abstract

This review summarizes novel taxon designations ascribed to prokaryotes derived from human primary clinical material during calendar year 2025, as well as proposed revisions to existing taxonomy. Major activity took place in the *Streptococcus* genus, as more than one dozen novel species were validly and effectively published, with two of these later classified as synonyms of *Streptococcus thalassemiae*. Moreover, whole genome sequencing and phylogenetic investigation of *Streptococcus mitis* group organisms resulted in a proposal to designate five species-level *Streptococcus* spp. taxa as *Streptococcus mitis* and an additional taxon as *Streptococcus oralis* subsp. *dentisani*. More than one dozen taxa were newly included in order *Enterobacterales* in 2025. Select novel taxa within genera *Providencia* and *Enterobacter* commonly possessed genotypes that conferred resistance to carbapenem and/or higher-generation cephem agents. *Stenotrophomonas muris* sp. nov. and *Terrisporobacter muris* sp. nov., initially characterized in gnotobiotic murine systems within the past 4 years, had clinical significance in human infection that was demonstrated in primary literature. The vast majority of the more than 70 novel obligate anaerobic taxa were derived from microbiome studies and had little ascribed clinical significance. Four novel obligate anaerobic Gram-negative taxa were shown to be of greater abundance in persons with Parkinson’s disease than in those without. Updates to taxa previously published in the *Journal of Clinical Microbiology* compendia reveal that several could serve as reservoirs for multiple antimicrobial resistance determinants. One example is the non-glucose fermentative Gram-negative bacillus *Pseudomonas juntendi*.

## INTRODUCTION

Central to the *Journal of Clinical Microbiology* publication series on novel and revised prokaryotic taxonomy relative to human primary specimens has been provision of data for general knowledge, pedagogy, and integration (if necessary) into routine clinical laboratory practice. This goal is also held by authors of past reviews of taxonomic elements relative to the fields of medical virology (1), parasitology (2), mycobacteriology (3), mycology (4), and bacteriology (5). Discussion of salient topics, as well as provision of additional clinical information (if found in primary publication) relative to important proposals and potentially highly-significant organisms, intends to edify this main objective (rather than becoming a narrative). Publication of bacterial taxonomy reviews on an annual basis for the past three years (5–7) has become consistent with a recent Clinical and Laboratory Standards Institute (CLSI) guideline (8) for implementation of important revised and novel taxonomy into routine medical microbiology laboratory practice.

Advances in whole genome and metagenomic sequencing, as especially applied to gastrointestinal foci of the human microbiome, have resulted in discoveries of multitudes of novel taxa. In fact, 146 nomenclatural elements of interest published and/or included in 2025 are presented in the following review. This datum includes 129 novel taxa (increased from an average of 78 novel taxa over the past three years [5–7]). Additional molecular-based characterizations frequently provide an impetus for reclassification of existing taxa. Knowledge of these novel or clarified taxonomic designations, some of which exhibit resistance to two or more classes of antimicrobial agents, may allow for increased understanding of pathogenesis in humans and subsequent contribution to clinical presentation. Moreover, other lines of research have begun to elucidate a role for selected bacteria in non-infectious conditions.

## MATERIALS AND METHODS

Effectively published taxa are those novel microbial species that have undergone polyphasic characterization and have been demonstrated not to possess a synonym taxon. Per the International Committee on Systematics of Prokaryotes (ICSP), validly published novel and revised taxa pertinent to prokaryotic species must satisfy requirements as set forth by The International Code of Nomenclature of Prokaryotes (ICNP) (9). Formal validation of a taxon makes the name available for use in the nomenclature of domains *Bacteria* and *Archaea*. Without such validation, a designation may exist in the literature as an effective publication but would not be recommended for use if following the rules of the ICNP.

One fulfillment for valid publication involves a properly-conscripted Latin binomial name and descriptive features of a novel species being published in the *International Journal of Systematic and Evolutionary Microbiology* (*IJSEM*). Required information must also include proof of deposit of type strains into recognized cultured collections located in two separate nations. An example relative to the Gram-negative bacillus *Stenotrophomonas forensis* is provided in reference 10. As of January 2018, taxon descriptions in *IJSEM* require that description of the 16S rRNA gene (or other taxonomically relevant genes) sequence accession number relative to the type strain be deposited in GenBank (11), with genome accession number included as part of the effective description. Similarity of this sequence is to be assessed versus related taxa.

As an alternative to primary publication in *IJSEM*, studies may be published in other journals and later included on a Validation List in *IJSEM* if specific requirements for valid publication of new names have been met. One past example relative to bacteria derived from clinical material is the effective description of the Gram-positive coccus *Streptococcus humanilactis* sp. nov. (12), with subsequent inclusion on an *IJSEM* Validation List (13). To be considered for inclusion on a Validation List (published six times per year), authors must submit a copy of the previously-published manuscript to the editorial office of *IJSEM* for confirmation that all elements necessary for valid publication (including culture collections) have been met. It has been estimated that one-half of effectively described, but non-*IJSEM*-published, taxa are not submitted to *IJSEM* for inclusion on Validation Lists (14).

Non-*IJSEM* journals that have recently published studies providing effective descriptions of human-derived novel taxa pertinent to this report, which may be relevant to the practice of medical microbiology, include *Anaerobe*, *Antonie Van Leeuwenhoek*, *Applied and Environmental Microbiology*, *Archives of Microbiology*, *Current Microbiology*, *European Journal of Clinical Microbiology and Infectious Diseases*, *iMeta*, *International Journal of Antimicrobial Agents*, *International Journal of Medical Microbiology*, *ISME Communications*, *Journal of Microbiology*, *MicrobiologyOpen*, *Microbiology Research*, *Microbiology Spectrum*, *Microorganisms*, *mSphere*, *Nature Communications*, *New Microbes and New Infections*, *Scientific Reports*, and *Standards in Genomic Sciences*. Journals that have recently published studies reflecting revisions in prokaryotic taxonomy relative to human clinical material include *Antonie Van Leeuwenhoek* and *Current Microbiology*.

A taxon validly published following an effective description in *IJSEM* or by inclusion on a Validation List may later be identified as a synonym (i.e., ascertainment of an identical organism with an antecedent taxonomic designation). Moreover, Trujillo et al. (15) stated that once a taxon is validly published or included on a Validation List, it remains available for use as long as both it is the correct name for the appropriate circumscription, position, and rank, and it is not subsequently rejected (*nomina rejicienda*) through a formal rejection process in accordance with ICNP. Organisms that are transferred to another taxon frequently do not relinquish a validly-published status; the majority of prokaryotic nomenclature revisions are due to changes in taxonomic opinion. As an example, the spirochete *Borreliella garinii* (16) currently remains effectively (and validly) published as a synonym to its antecedent (and correct) name *Borrelia garinii* (17). Table headers throughout this document include phrases, such as “Earlier Validly Published Name” and “2025 Validly Published Name” to reflect this paradigm. The protocols outlined in this paragraph align, in part, with a position paper (18) recently published by an *ad hoc* committee of ICSP that has assumed the charge of mitigating the impact of nomenclature changes.

All issues of *IJSEM* published from January 2025 through December 2025 (including six Validation Lists) were manually searched for original articles describing novel, validly published species taxonomy or included changes in taxonomic nomenclature. With the goal of consistency, only nomenclature with taxonomic status of “correct name” (per data curated by LPSN—List of Prokaryotic Names with Standing in Nomenclature; https://Lpsn.dsmz.de) was included. On rare occasion, taxa accepted in *IJSEM* during calendar year 2025 (e.g., the Gram-negative coccus [pairs or small clumps] *Moraxella veridica* sp. nov. [19]) were subsequently classified by LPSN as synonyms (e.g., correct name *Moraxella catarrhalis* [20]) and were thus excluded. (Validation Lists have also been the source of apparent post-publication reversion to synonym status; *Teichococcus cervicalis* comb. nov [21, 22], subsequently being deferred to its correct and non-synonym designation of *Roseomonas cervicalis* [23, 24] is presented as one example). The audit was further filtered by organisms recovered from human clinical material. When an initial organism reservoir could not be ascertained, PubMed primary literature searches (United States National Library of Medicine and the National Institutes of Health) attempted to index subsequent literature for further investigation.

Some primary publications simply identified isolates as being derived from a specific specimen source (including traditional sterile body sites) but did not provide contextual clinical data. A number of novel taxonomy investigations were initiated on archival isolates or carried out by reference laboratories who may not have had access to extensive clinical data. In such scenarios, clinical significance of these taxa was interpreted as “Not established.” Moreover, a number of novel taxa included within this compendium, inclusive of two novel species of *Corynebacterium* (25, 26), were derived from blood cultures but were not accompanied by summaries of clinical manifestations or documentation of laboratory data that could suggest an acute inflammatory process. For three additional Gram-negative novel taxa (27), an anatomic source was indicated that would have likely clinical significance if aspiration were utilized during specimen collection, while cutaneous swab collections may result in uncertainty within the realm of commensal flora; however, such data regarding means of specimen acquisition were not published. Paucities of provided clinical data relegate pathogenicity and clinical significance assessments of novel taxa discoveries to debate. Additional studies and published case reports (28, 29) from reliable journal outlets (30) can assist in the further clinical characterization of novel taxa.

## RESULTS AND DISCUSSION

Compilations of novel taxa recovered from human sources stratified by Gram morphology and oxygen growth requirement (when fully provided) are presented in Tables 1 to 5, with the phenotypic means of taxa organization within Tables 1 to 4 geared toward the practicing medical microbiologist. Organisms are presented in alphabetic order within each stratification. Table 6 provides taxonomic revision proposals for organisms originally or subsequently recovered from human sources. On the basis of recent peer-reviewed publications, Table 7 attempts to ascribe clinical relevance to or provide updates for a number of taxa whose significance was inconclusive in previous *Journal of Clinical Microbiology* compendia (5–7, 31–34). Important findings are discussed later in this section.

**TABLE 1:** Novel, validly published, aerobic/facultative Gram-positive bacterial species recovered from human clinical material reported from January 2025 through December 2025

Scientific name	Family	Source^*a*^	Clinical relevance	Growth characteristics	Reference(s)
Gram-positive cocci					
*Enterococcus immobilis* sp. nov.	*Enterococcaceae*	Feces from a human with rheumatoid arthritis	Not established	Limited phenotypic characterization provided in reference 35; Gram reaction and atmospheric requirement predicted by genus assignment	(35)^*b*^
*Enterococcus infantis* sp. nov.	*Enterococcaceae*	Feces from an infant with atopic dermatitis in Taiwan	Not established	Facultative, catalase-negative Gram-positive coccus; 0.3- to 0.6-mm diameter circular, smooth, α-hemolytic colonies on tryptic soy agar with 5% sheep blood at 37°C in 1% NaCl; does not tolerate 6% NaCl; positive for glycerol, L-arabinose, melibiose, sucrose, melezitose, alkaline phosphatase, valine arylamidase, cystine arylamidase; negative for naphthol-AS-BI-phosphohydrolase, 5-keto-gluconate	(36)
*Macrococcus psychrotolerans* sp. nov.	*Staphylococcaceae*	Archival isolates (2) from nasal swab and pus on leg wound in Czech Republic	Not established	Aerobic, non-motile, non-spore-forming, catalase-positive, oxidase-positive Gram-positive coccus (clusters and tetrads); 2-mm diameter beige, circular, flat, smooth colonies on tryptone soy agar at 1°C–45°C in up to 10% NaCl; non-hemolytic on blood agar; growth on mannitol salt agar; resistant to bacitracin; positive for pyrrolidonyl arylamidase, glycerol (some stains weakly positive), ribose; negative for galactose, lactose, mannitol; variable reactions for Voges-Proskauer, nitrate reduction; possesses plasmids harboring *mecB* determinant	(37)^*c*^
*Streptococcus dentalis* sp. nov.	*Streptococcaceae*	Saliva	Not established	Facultative, non-motile, catalase-negative, oxidase-negative Gram-positive coccus; 0.5- to 2.0-mm diameter pale white, circular, umbonate, opaque colonies on Columbia agar; optimal growth at 37°C in 0%–2% NaCl (optimum 0%); positive for glycyl-tryptophan arylamidase; negative for alkaline phosphatase, leucine aminopeptidase, β-galactosidase, α-galactosidase, D-raffinose, D-maltose; resistant to azithromycin, tetracycline, gentamicin; susceptible to clindamycin, ampicillin, imipenem, moxifloxacin, vancomycin	(38)^*b*^
*Streptococcus fermentans* sp. nov.	*Streptococcaceae*	Breast milk from healthy women in China	Not established	Facultative, non-spore-forming, catalase-negative Gram-positive coccus; 0.55-mm diameter white, circular, smooth, opaque, convex colonies on M17 agar; γ-hemolysis on brain heart infusion (BHI) agar with sheep blood; optimal growth at 37°C–40°C in 0.0%–0.2% NaCl; positive for β-galactosidase, alkaline phosphatase, leucine aminopeptidase, lactose; negative for starch, acetoin, pyrrolidone aromatase, trehalose, glycogen	(39)
*Streptococcus gingivalis* sp. nov.	*Streptococcaceae*	Saliva	Not established	Facultative, non-motile, catalase-negative, oxidase-negative Gram-positive coccus; 0.5- to 2.0-mm diameter pale white, circular, umbonate, opaque colonies on Columbia agar; optimal growth at 37°C in 0%–2% NaCl (optimum 0%); positive for glycyl-tryptophan arylamidase, alkaline phosphatase, β-galactosidase; negative for leucine aminopeptidase, α-galactosidase, D-raffinose, D-maltose; resistant to azithromycin, tetracycline, gentamicin; susceptible to clindamycin, ampicillin, imipenem, moxifloxacin, vancomycin	(38)^*b*^
*Streptococcus immobilis* sp. nov.	*Streptococcaceae*	Feces from humans with rheumatoid arthritis	Not established	Limited phenotypic characterization provided in reference 35; Gram reaction and atmospheric requirement predicted by genus assignment	(35)^*b*^
*Streptococcus jiangnanensis* sp. nov.	*Streptococcaceae*	Breast milk from healthy women in China	Not established	Facultative, non-spore-forming, catalase-negative Gram-positive coccus; 0.45-mm diameter white, circular, smooth, opaque, convex colonies on M17 agar; γ-hemolysis on BHI agar with sheep blood; optimal growth at 37°C–40°C in 0.0%–0.2% NaCl; positive for acetoin, trehalose, starch, β-galactosidase, alkaline phosphatase, leucine aminopeptidase, lactose; negative for pyrrolidone aromatase, glycogen	(39)
*Streptococcus lingualis* sp. nov.	*Streptococcaceae*	Saliva	Not established	Facultative, non-motile, catalase-negative, oxidase-negative Gram-positive coccus; 0.5- to 2.0-mm diameter pale white, circular, umbonate, opaque colonies on Columbia agar; optimal growth at 37°C in 0%–3% NaCl (optimum 0%); positive for glycyl-tryptophan arylamidase, alkaline phosphatase, leucine aminopeptidase, α-galactosidase, D-raffinose, D-maltose; negative for β-galactosidase; resistant to tetracycline, gentamicin; susceptible to azithromycin, clindamycin, ampicillin, imipenem, moxifloxacin, vancomycin	(38)^*b*^
*Streptococcus mitis* subsp. *mitis* subsp. nov.	*Streptococcaceae*	Archival isolates of oral cavity viridans group *Streptococcus* spp.	Not established	Facultative, non-motile, non-spore-forming, catalase-negative Gram-positive coccus; α-hemolytic on blood agar; susceptible to optochin; several strains produce a capsular polysaccharide identical to that found in selected serotypes of *Streptococcus pneumoniae*; positive for glucose, sucrose; negative for esculin, arginine, inulin, sorbitol, mannitol; the published taxon *Streptococcus mitis* subsp. *carlssonii* subsp. nov, (40) has subsequently been classified as illegitimate	(40)
*Streptococcus nakanonensis* sp. nov.	*Streptococcaceae*	Sputum from a patient in Japan	Initially identified as *Streptococcus pneumoniae* in a hospitalized patient with pneumonia	Initially published as *Streptococcus nakanoensis*; facultative, non-spore-forming, catalase-negative Gram-positive coccus; 0.25- to 0.5-mm diameter α-hemolytic colonies on Columbia agar with 5% sheep blood in ambient conditions; optimal growth at 20°C–37°C; does not possess Lancefield antigens; susceptible to optochin in ambient and capnophilic conditions; exhibits bile solubility; positive for D-ribose, D-trehalose, D-raffinose; negative for α-galactosidase, alkaline phosphatase, β-galactosidase, glycyl-tryptophan arylamidase; refer to Table 2 for subsequent taxonomic revision	(41)^*d*^
*Streptococcus parapneumoniae* sp. nov.	*Streptococcaceae*	Blood from a patient in Japan	Patient with bacteremia and pyelonephritis	Facultative, non-spore-forming, catalase-negative Gram-positive coccus; 0.4- to 0.8-mm diameter α-hemolytic colonies on Columbia agar with 5% sheep blood in ambient conditions; optimal growth at 20°C–37°C; does not possess Lancefield antigens; positive for L-leucine-β-naphthylamide, inulin, starch hydrolysis, D-raffinose; negative for pyroglutamic acid-β-naphthylamide, D-trehalose, esculin, alkaline phosphatase, acid phosphatase, α-galactosidase; resistant to levofloxacin, erythromycin, clindamycin; susceptible to ceftriaxone, meropenem, vancomycin, linezolid; intermediate susceptibility to penicillin; refer to Table 2 for subsequent taxonomic revision	(42)^*d*^
*Streptococcus taonis* sp. nov.	*Streptococcaceae*	Blood from a pediatric patient in Taiwan	Suspected bloodstream infection in child with fever and croup	Facultative, non-spore-forming, catalase-negative Gram-positive coccus; 0.5- to 1.0-mm diameter circular, smooth colonies on blood agar at 37°C in 0%–2% NaCl; positive for galactose, acetyl glucosamine, lactose; negative for ribose, melibiose, trehalose, D-raffinose, α-fucosidase, α-galactosidase	(43)^*c*^
*Streptococcus taoyuanensis* sp. nov.	*Streptococcaceae*	Blood from a pediatric patient in China	Croup and radiologic findings of bronchopneumonia	Facultative, non-spore-forming, catalase-negative, oxidase-negative Gram-positive coccus; 0.5- to 1.0-mm diameter circular, smooth, α-hemolytic colonies on BHI blood agar; optimal growth at 37°C in 0.0%–0.5% NaCl; non-groupable with Lancefield A, B, C, D, F, G antisera; positive for melibiose, sorbitol, alkaline phosphatase, esterase, β-galactosidase; negative for amygdalin, mannitol, trehalose, cellobiose; resistant to clindamycin, erythromycin; susceptible to cefotaxime, vancomycin	(44)^*e*^
*Streptococcus vaginalis* sp. nov.	*Streptococcaceae*	Vaginal swab of a pregnant woman in Taiwan with type 2 diabetes	Not established; isolated derived in context of prenatal *Streptococcus agalactiae* screening	Facultative, non-spore-forming Gram-positive coccus; colonies on BHI agar with 5% sheep blood demonstrate γ-hemolysis at 30°C–45°C; tolerates up to 5.5% NaCl; positive for amygdalin, melibiose, D-raffinose, β-gentiobiose; negative for C4 esterase, β-galactosidase, α-glucosidase	(45)^*c*^
*Streptococcus vulneris* sp. nov.	*Streptococcaceae*	Wound of a patient with diabetic foot ulcer in Taiwan	Not established	Facultative, non-spore-forming, catalase-negative Gram-positive coccus; α-hemolytic colonies on BHI agar with 5% sheep blood at 37°C; failed to grow in 2% NaCl; positive for galactose, acetyl glucosamine, β-galactosidase; negative for melibiose, ribose, trehalose, D-raffinose	(46)^*e*^
*Streptococcus wuxiensis* sp. nov.	*Streptococcaceae*	Breast milk from healthy women in China	Not established	Facultative, non-spore-forming, catalase-negative Gram-positive coccus; 0.45-mm diameter white, circular, smooth, opaque, convex colonies on M17 agar; γ-hemolysis on BHI agar with sheep blood; optimal growth at 37°C–40°C in 0%–1% NaCl; positive for β-galactosidase, leucine aminopeptidase, pyrrolidone aromatase, lactose; negative for glycogen, acetoin, trehalose, starch, alkaline phosphatase	(39)
Gram-positive bacilli					
*Bacillus phocaeensis* sp. nov.	*Bacillaceae*	Feces from a boy in Senegal with kwashiorkor	Not established	Aerobic, motile, spore-forming, catalase-positive, oxidase-negative Gram-positive bacillus; 0.5- to 0.8-mm diameter gray, circular, smooth colonies on Columbia agar with 5% sheep blood; optimal growth at 37°C; positive for D-glucose, D-fructose, D-maltose, D-lactose; negative for nitrate reductase, urease, β-galactosidase, ribose, mannose, mannitol	(47)^*c*^
*Corynebacterium haematomassiliense* sp. nov.	*Corynebacteriaceae*	Blood from a male with chronic obstructive pulmonary disease in France	Not established	Initially published as *Corynebacterium haemomassiliense*; facultative, non-motile, non-spore-forming, catalase-positive, oxidase-negative Gram-positive bacillus; white, non-hemolytic colonies on Columbia agar with 5% sheep blood; optimal growth at 31.5°C–56°C in 1.0%–1.5% NaCl; positive for alkaline phosphatase, *N*-acetylglucosamine, D-ribose, D-glucose; negative for L-arabinose, D-mannose, D-maltose	(25)^*b*^
*Corynebacterium mayonis* sp. nov.	*Corynebacteriaceae*	Blood from a patient in the United States; isolate characterized by reference laboratory	Not established	Facultative, non-motile, catalase-positive, oxidase-negative Gram-positive bacillus; white, opaque, non-hemolytic colonies with halo-like edges on tryptic soy agar with 5% sheep blood at 35°C; robust growth in presence of 0%–6% NaCl; positive for alkaline phosphatase, pyrazinamidase, D-glucose; negative for pyrrolidonyl arylamidase, nitrate reductase, urease, gelatin hydrolysis, D-ribose, D-maltose; susceptible to penicillin, ciprofloxacin, gentamicin, vancomycin, tetracycline, meropenem; intermediate susceptibility to erythromycin, clindamycin; partial 16S rRNA sequencing indicated closest match to *Corynebacterium auris*	(26)
*Corynebacterium neomassiliense* sp. nov.	*Corynebacteriaceae*	Feces from a healthy male pygmy in France	Not established	Facultative, non-motile, non-spore-forming, catalase-positive, oxidase-negative Gram-positive bacillus; white, punctiform colonies on Columbia agar with 5% sheep blood; optimal growth at 37°C in 5% NaCl; positive for urease, alkaline phosphatase, C8 esterase lipase, D-fructose; negative for nitrate reductase, β-galactosidase, D-arabinose, mannose, mannitol	(48)^*f*^
*Corynebacterium rhinophilum* sp. nov.	*Corynebacteriaceae*	Isolates (12) from nasal or nasopharyngeal swabs collected in United States and Botswana	Not established	Aerobic (and microaerophilic), non-motile, non-spore-forming, catalase-positive, oxidase-negative Gram-positive bacillus; 2- to 4-mm diameter creamy white, raised or umbonate colonies on BHI agar with 1% Tween 80; optimal growth at 37°C in up to 7.5% NaCl (5% for some strains); positive for alkaline phosphatase; 40% of strains positive for nitrate reductase; 60% of strains positive for pyrrolidonyl arylamidase; negative for gelatin hydrolysis, esculin hydrolysis, lactose, maltose, sucrose, glycogen	(49)
*Robertmurraya yapensis* sp. nov.	*Cytobacillaceae*	Human feces	Not established	Phenotypic characterization not provided	(50)^*c*^
*Scrofimicrobium appendicitidis* sp. nov.	*Actinomycetaceae*	Peritoneal swab from a patient in Hong Kong	Ruptured appendix, abscess, gangrene	Facultative, non-motile, catalase-negative, oxidase-negative Gram-positive bacillus; 0.5- to 1.0-mm diameter gray, semi-translucent, low convex colonies on blood agar at 37°C; positive for urease, leucine arylamidase, tyrosine arylamidase, phenylalanine arylamidase D-xylose, pyruvate; negative for indole, sucrose, D-maltose, arbutin, esculin, arginine	(51)
*Tessaracoccus massiliensis* sp. nov.	*Propionibacteriaceae*	Feces from a Nigerian child with kwashiorkor	Not established	Facultative, non-motile, non-spore-forming, catalase-positive, oxidase-negative Gram-positive bacillus; 0.7- to 1.1-mm diameter gray, circular, shiny, smooth colonies on Columbia agar with 5% sheep blood; optimal growth at 37°C; growth in 0%–5% NaCl (optimum, 0%); positive for nitrate reductase, β-galactosidase, L-arabinose, D-mannose, D-glucose; negative for D-mannitol, D-maltose	(52)^*c*^

^*a*^ Number of isolates from clinical source is 1, except where shown in parentheses.

^*b*^ Validly published by inclusion in Validation List no. 223 (53).

^*c*^ Validly published by inclusion in Validation List no. 225 (54).

^*d*^ Validly published by inclusion in Validation List no. 221 (22).

^*e*^ Validly published by inclusion in Validation List no. 224 (55).

^*f*^ Validly published by inclusion in Validation List no. 222 (56).

**TABLE 2:** Novel, validly published, aerobic/facultative Gram-negative bacilli recovered from human clinical material reported from January 2025 through December 2025

Scientific name	Family	Source^*a*^	Clinical relevance	Growth characteristics	Reference(s)
*Capnocytophaga endodontalis* sp. nov.	*Flavobacteriaceae*	Left mandibular second molar area from a patient in Republic of Korea	Refractory periapical abscess	Gram-negative fusiform bacillus with gliding motility; 0.8-mm diameter colonies on tryptic soy agar with yeast extract, cysteine HCl-H_2_O, hemin, and vitamin K1 supplements in anaerobic conditions (aerobic characterization not attempted); optimal growth at 35°C in 1% NaCl; positive for esculin hydrolysis, D-cellobiose, D-glucose, D-lactose, D-sucrose; negative for indole, nitrate reductase, D-mannitol, D-xylose	(57)^*b*^
*Dryocola baronae* sp. nov.	*Enterobacteriaceae*	Knee from a patient in the United States	Not established	Facultative, motile, oxidase-negative, catalase-negative Gram-negative bacillus; white/cream-colored, smooth, opaque, non-hemolytic colonies on tryptic soy agar with 5% sheep blood; growth at 30°C–37°C in up to 10% NaCl; positive for glycerol, D-maltose, D-raffinose; negative for D-adonitol, D-arabitol, arginine dihydrolase; susceptible to gentamicin, ciprofloxacin, meropenem; resistant to ampicillin, cefazolin	(27)
*Dryocola mayonis* sp. nov.	*Enterobacteriaceae*	Knee from a patient in the United States	Not established	Facultative, motile, oxidase-negative, catalase-negative Gram-negative bacillus; white/cream-colored, smooth, opaque, non-hemolytic colonies on tryptic soy agar with 5% sheep blood; growth at 30°C–37°C in up to 10% NaCl; positive for glycerol, D-maltose, D-adonitol, D-arabitol, arginine dihydrolase; negative for D-raffinose; susceptible to gentamicin, ampicillin, ciprofloxacin, meropenem	(27)
*Dryocola sharpae* sp. nov.	*Enterobacteriaceae*	Foot from a patient in the United States	Not established	Facultative, motile, oxidase-negative, catalase-negative Gram-negative bacillus in small aggregates; white/cream-colored, smooth, opaque, non-hemolytic colonies on tryptic soy agar with 5% sheep blood; growth at 30°C–37°C in up to 10% NaCl; positive for D-adonitol, D-arabitol; negative for glycerol, D-raffinose, D-maltose, arginine dihydrolase; susceptible to gentamicin, ampicillin, ciprofloxacin, meropenem; intermediate susceptibility to cefazolin	(27)
*Enterobacter chinensis* sp. nov.	*Enterobacteriaceae*	Blood from a patient in China	Patient in the case series (58) was febrile and immunocompromised due to chemotherapy-induced neutropenia and/or renal transplantation immunosuppressive agents	Facultative, motile, oxidase-negative, catalase-positive Gram-negative bacillus; white, round, translucent, convex, smooth colonies on nutrient agar; optimal growth at 37°C; positive for ornithine decarboxylase, citrate, Voges-Proskauer, ONPG, gelatinase, D-turanose; negative for arginine dihydrolase, lysine decarboxylase, L-rhamnose; susceptible to gentamicin, aztreonam, piperacillin-tazobactam, imipenem, ceftriaxone, cefepime, ciprofloxacin, tetracycline; resistant to cefazolin, amoxicillin-clavulanate, colistin, trimethoprim-sulfamethoxazole; intermediate susceptibility to ampicillin	(58)^*c*^
*Enterobacter intestinihominis* sp. nov.	*Enterobacteriaceae*	Human feces	Not established in reference 50; two urinary tract isolates with advanced cephem resistance discussed in reference 59	Phenotypic characterization not provided; Gram reaction and atmospheric requirement predicted by genus assignment	(50)^*d*^
*Enterobacter rongchengensis* sp. nov.	*Enterobacteriaceae*	Blood from a patient in China	Patient in the case series (58) was febrile and immunocompromised due to chemotherapy-induced neutropenia and/or renal transplantation immunosuppressive agents	Facultative, motile, oxidase-negative, catalase-positive Gram-negative bacillus; white, circular, translucent, convex, smooth colonies on nutrient agar; optimal growth at 37°C in up to 9% NaCl; positive for arginine dihydrolase, ornithine decarboxylase, citrate, Voges-Proskauer, ONPG, gelatinase, L-rhamnose; negative for lysine decarboxylase, D-turanose; susceptible to gentamicin, aztreonam, piperacillin-tazobactam, imipenem, ceftriaxone, cefepime, ciprofloxacin, tetracycline; resistant to cefazolin, amoxicillin-clavulanate, ampicillin, ampicillin-sulbactam, cefotaxime, trimethoprim-sulfamethoxazole; intermediate susceptibility to ceftazidime, colistin	(58)^*c*^
*Mixta hanseatica* sp. nov.	*Erwiniaceae*	Blood from an elderly male patient in Germany	Sepsis potentially secondary to cellulitis	Facultative, motile, oxidase-negative, catalase-positive Gram-negative bacillus; non-hemolytic, mucoid, gray colonies with depressed center on Columbia sheep blood agar at 37°C; positive for tryptophan deaminase, Voges-Proskauer, gelatinase, nitrate reduction; negative for ONPG, arginine dihydrolase, ornithine decarboxylase, lysine decarboxylase, citrate, urease, indole, H_2_S; resistant to ampicillin, piperacillin; susceptible to ampicillin-sulbactam, ceftriaxone, ceftazidime, imipenem, ciprofloxacin, tobramycin, trimethoprim-sulfamethoxazole	(60)^*b*^
*Proteus appendicitidis* sp. nov.	*Morganellaceae*	Pus	Patient with appendicitis in China	Facultative Gram-negative bacillus; swarming colonies observed on Luria-Bertani (LB) agar; small, translucent, neat-edged circular colonies observed when cultures were incubated in anaerobic conditions; optimal growth at 30°C in 1% NaCl; positive for methyl-α-D-glucopyranoside, indole; negative for citrate, D-fructose, L-rhamnose, melezitose, turanose, salicin; resistant to ampicillin, cefazolin, cefuroxime, nitrofurantoin	(61)^*b*^
*Providencia hangzhouensis* sp. nov.	*Morganellaceae*	Urine from a patient in China	Urinary tract infection	Facultative, motile, oxidase-negative Gram-negative bacillus; yellow, circular, raised, opaque, smooth colonies on nutrient agar at 37°C; positive for indole, urease, Voges-Proskauer, citrate, lysine deaminase; negative for lysine decarboxylase, arginine dihydrolase, ornithine decarboxylase, gelatinase, hydrogen sulfide, melibiose; *bla*_IMP-27_ most prevalent carbapenemase determinant	(62)^*c*^
*Providencia xianensis* sp. nov.	*Morganellaceae*	Isolates (2) from China; one specifically noted from ascites fluid	Two patients from separate locations with septic shock	Facultative, motile, oxidase-negative, catalase-positive Gram-negative bacillus; yellow, raised, opaque, circular, smooth colonies on tryptic soy agar; optimal growth at 37°C in up to 7% NaCl; positive for urease, glycerol, citrate, lysine deaminase, D-glucose, D-mannitol, arbutin; negative for arginine dihydrolase, ornithine decarboxylase, lysine decarboxylase, H_2_S, indole, Voges Proskauer, gelatinase, β-galactosidase, sucrose, melibiose; resistant to ampicillin, ceftazidime, imipenem, tetracycline, colistin, trimethoprim-sulfamethoxazole, ciprofloxacin; susceptible to aztreonam, amikacin; variable susceptibility to gentamicin, cefepime, meropenem	(63)^*c*^
*Providencia xihuensis* sp. nov.	*Morganellaceae*	Blood from a patient in China	Pancreatic mass, followed by several surgical interventions	Facultative, non-motile, oxidase-negative, catalase-positive Gram-negative bacillus; yellow, circular, smooth colonies on Columbia blood agar; optimal growth at 37°C in up to 7% NaCl; positive for citrate, lysine deaminase, indole, D-glucose, D-mannitol, urease, L-rhamnose, esculin, arbutin, salicin; negative for D-lyxose; resistant to ampicillin, ciprofloxacin, tetracycline, trimethoprim-sulfamethoxazole, colistin, polymyxin B, tetracycline; susceptible to ceftazidime, cefepime, imipenem, meropenem, gentamicin, aztreonam	(64)^*e*^
*Providencia zhejiangensis* sp. nov.	*Morganellaceae*	Feces from a patient in China	Elevated C-reactive protein	Facultative, non-motile, oxidase-negative, catalase-positive Gram-negative bacillus; yellow, opaque, circular, raised, smooth colonies on Columbia blood agar; optimal growth at 37°C in up to 7% NaCl; positive for citrate, lysine deaminase, indole, D-glucose, D-lyxose; negative for D-mannitol, urease, L-rhamnose, esculin, arbutin, salicin; resistant to ceftazidime, cefepime, imipenem, meropenem, gentamicin, ampicillin, ciprofloxacin, tetracycline, trimethoprim-sulfamethoxazole, colistin, polymyxin B, tetracycline; susceptible to aztreonam	(64)^*e*^
*Providencia zhijiangensis* sp. nov.	*Morganellaceae*	Bile from a patient in China	Acute obstructive suppurative cholangitis and septic shock	Facultative, motile, oxidase-negative Gram-negative bacillus; yellow, raised, opaque, circular, smooth colonies on nutrient agar; optimal growth at 35°C–37°C; positive for Voges-Proskauer, phenylalanine deaminase, citrate, sucrose, D-mannitol, glycerol; negative for arginine dihydrolase, ornithine decarboxylase, lysine decarboxylase, urease, indole, H_2_S, β-galactosidase; *bla*_IMP-27_ and *bla*_NDM-1_ most prevalent carbapenemase determinants	(65)^*c*^
*Pseudomonas pharyngis* sp. nov.	*Pseudomonadaceae*	Throat swab from a patient in Japan	Not established	Aerobic, motile, non-spore-forming, oxidase-positive, catalase-positive Gram-negative bacillus; 1- to 5-mm diameter creamy, convex, circular colonies on LB agar; growth at 4°C–36°C in 0%–6% NaCl; fluorescent pigments on King’s B agar; positive for L-arginine, gelatin hydrolysis, D-mannose, C4 esterase, C8 esterase lipase; negative for urease, esculin hydrolysis, maltose, adipic acid, C14 lipase, D-galacturonic acid, D-glucuronic acid; weakly positive for Tween 40 hydrolysis, C14 lipase	(66)^*d*^
*Pseudomonas sputi* sp. nov.	*Pseudomonadaceae*	Sputum from a patient in Japan	Not established	Aerobic, motile, non-spore-forming, oxidase-positive, catalase-positive Gram-negative bacillus; 1- to 3-mm diameter creamy, convex, circular colonies on LB agar; growth at 4°C–36°C in 0-6% NaCl; fluorescent pigments on King’s B agar; positive for L-arginine, gelatin hydrolysis, D-mannose, C4 esterase, C8 esterase lipase, D-galacturonic acid, D-glucuronic acid; negative for urease, esculin hydrolysis, maltose, adipic acid, C14 lipase, Tween 40 hydrolysis	(66)^*b*^
*Roseomonas cutis* sp. nov.	*Acetobacteraceae*	Skin swab specimen (behind ear) from a male in South Korea	Not established	Aerobic, motile, oxidase-positive, catalase-positive Gram-negative bacillus; 0.5- to 1.0-mm diameter pink-pigmented, circular, convex, smooth, entire colonies on nutrient agar (orange, sticky colonies on tryptic soy agar); optimal growth at 28°C in up to 3% NaCl; positive for nitrate reductase, sodium maleate, valine arylamidase, acid phosphatase, DNase, urease, starch hydrolysis; negative for D-glucose, glycerol, lactose, sodium acetate, leucine arylamidase, α-glucosidase	(67)
*Skermanella cutis* sp. nov.	*Azospirillaceae*	Forehead skin swab from male volunteers	Not established	Microaerophilic, motile, oxidase-positive, catalase-positive Gram-negative bacillus; 3- to 6-mm diameter pale-pink, translucent, mucoid, circular colonies on nutrient agar; optimal growth at 25°C–30°C in 0.5%–1.0% NaCl; positive for D-arabinose, D-glucose, D-mannitol, D-arabitol, alkaline phosphatase, C4 esterase, C8 esterase lipase; negative for D-maltose, D-melibiose, gentiobiose, glycerol, inulin, D-trehalose	(68)^*f*^
*Stenotrophomonas muris* sp. nov.	*Lysobacteraceae*	Blood, sputum	Fever, chills, rigors in one patient post-amputation; acute lymphoblastic leukemia with hemorrhagic pneumonia in a second patient	Initial report of isolation from gastrointestinal tract of specific pathogen-free mouse published in reference 69 and accepted in Validation List no. 221 (22); limited phenotypic characterization provided; Gram reaction and atmospheric requirement predicted by genus assignment	(70)

^*a*^ Number of isolates from clinical source is 1, except where shown in parentheses.

^*b*^ Validly published by inclusion in Validation List no. 226 (71).

^*c*^ Validly published by inclusion in Validation List no. 221 (22).

^*d*^ Validly published by inclusion in Validation List no. 225 (54).

^*e*^ Validly published by inclusion in Validation List no. 224 (55).

^*f*^ Validly published by inclusion in Validation List no. 223 (53).

**TABLE 3:** Novel, validly published, obligate anaerobic bacterial species recovered from human clinical material reported from January 2025 through December 2025

Scientific name	Family	Source^*a*^	Clinical relevance	Growth characteristics	Reference(s)
Gram-positive anaerobes					
*Anaerococcus cruorum* sp. nov.	*Peptoniphilaceae*	Isolates (3) from blood in the Netherlands	≤50% of blood culture sets positive in each of the three patients	Anaerobic, catalase-negative, large Gram-positive coccus; white-gray, small colonies on Brucella blood agar at 37°C; resistant to colistin, susceptible to kanamycin, vancomycin (for identification purposes); positive for pyroglutamic acid arylamidase, mannose; negative for urease, α-glucosidase, arginine arylamidase, leucine arylamidase, raffinose; reported susceptibility to amoxicillin, metronidazole, clindamycin	(72)^*b*^
*Anaerococcus groningensis* sp. nov.	*Peptoniphilaceae*	Isolates (2) from blood in the Netherlands	Two patients with fever or bacteremia with unknown focus; two positive sets (sole isolate) of blood cultures in each patient	Anaerobic, catalase-negative, large Gram-positive coccus; white-gray, small colonies on Brucella blood agar at 37°C; resistant to colistin, susceptible to kanamycin, vancomycin (for identification purposes); positive for pyroglutamic acid arylamidase, mannose; negative for urease, α-glucosidase, arginine arylamidase, leucine arylamidase, raffinose; reported susceptibility to amoxicillin, metronidazole; 50% of isolates resistant to clindamycin	(72)^*b*^
*Anaerococcus kampingae* sp. nov.	*Peptoniphilaceae*	Isolates (2) from tissue, purulent material in the Netherlands	Not established; concomitant isolation of *Serratia marcescens* implicated as disease etiology in purulent material	Published in reference 72 as *Anaerococcus kampingiae*; anaerobic, catalase-negative, large Gram-positive coccus; white-gray, small colonies on Brucella blood agar at 37°C; resistant to colistin, susceptible to kanamycin, vancomycin (for identification purposes); positive for urease, α-glucosidase, arginine arylamidase, leucine arylamidase, mannose, raffinose; negative for pyroglutamic acid arylamidase; reported susceptibility to amoxicillin, metronidazole; 50% of isolates resistant to clindamycin	(72)^*b*^
*Anaerococcus martiniensis* sp. nov.	*Peptoniphilaceae*	Isolates (3) from thoracic debris, blood, ascites in the Netherlands	Sole isolate in two of the patients, one of whom was diagnosed with peritonitis carcinomatosis	Anaerobic, catalase-negative, large Gram-positive coccus; white-gray, small colonies on Brucella blood agar at 37°C; resistant to colistin, susceptible to kanamycin, vancomycin (for identification purposes); positive for pyroglutamic acid arylamidase, mannose; negative for urease, α-glucosidase, arginine arylamidase, leucine arylamidase, raffinose; reported susceptibility to amoxicillin, metronidazole, clindamycin	(72)^*b*^
*Anaerosinus massiliensis* sp. nov.	*Sporomusaceae*	Feces from young, healthy female in Senegal	Not established	Past designation of “*Massilibacillus massiliensis*” (73) from clinical material never validly published; anaerobic, catalase-negative, oxidase-negative Gram-positive bacillus; 2-mm diameter translucent colonies with white center on Columbia agar with 5% sheep blood; no additional phenotypic data provided	(74)
*Bifidobacterium hominis* sp. nov.	*Bifidobacteriaceae*	Human feces	Not established	Phenotypic characterization not provided; Gram reaction and atmospheric requirement predicted by genus assignment	(50)^*c*^
*Blautia aquisgranensis* sp. nov.	*Lachnospiraceae*	Human feces	Not established	Phenotypic characterization not provided; Gram reaction and atmospheric requirement predicted by genus assignment	(50)^*c*^
*Blautia arthritidis* sp. nov.	*Lachnospiraceae*	Feces from a human with rheumatoid arthritis	Not established	Limited phenotypic characterization provided in reference 35; Gram reaction and atmospheric requirement predicted by genus assignment	(35)^*d*^
*Blautia caccae* sp. nov.	*Lachnospiraceae*	Human feces	Not established	Phenotypic characterization not provided; Gram reaction and atmospheric requirement predicted by genus assignment	(50)^*c*^
*Blautia flagellata* sp. nov.	*Lachnospiraceae*	Feces from a human with rheumatoid arthritis	Not established	Limited phenotypic characterization provided in reference 35; Gram reaction and atmospheric requirement predicted by genus assignment	(35)^*d*^
*Blautia immobilis* sp. nov.	*Lachnospiraceae*	Feces from a human with rheumatoid arthritis	Not established	Limited phenotypic characterization provided in reference 35; Gram reaction and atmospheric requirement predicted by genus assignment	(35)^*d*^
*Blautia intestinihominis* sp. nov.	*Lachnospiraceae*	Human feces	Not established	Phenotypic characterization not provided; Gram reaction and atmospheric requirement predicted by genus assignment	(50)^*c*^
*Blautia longa* sp. nov.	*Lachnospiraceae*	Feces from a human with rheumatoid arthritis	Not established	Limited phenotypic characterization provided in reference 35; Gram reaction and atmospheric requirement predicted by genus assignment	(35)^*d*^
*Blautia ovalis* sp. nov.	*Lachnospiraceae*	Feces from a human with rheumatoid arthritis	Not established	Limited phenotypic characterization provided in reference 35; Gram reaction and atmospheric requirement predicted by genus assignment	(35)^*d*^
*Catenibacterium mitsuokai* subsp. *mitsuokai* subsp. nov.	*Coprobacillaceae*	Feces	Not established	Taxon created by characterization of *C. mitsuokai* subsp. *tridentinum* subsp. nov.; original isolation of *Catenibacterium mitsuokai* described in reference 75; anaerobic, non-motile, non-spore-forming Gram-positive bacillus; optimal growth at 37°C; optimal pH range 6–7; positive for leucyl-glycine arylamidase; negative for maltose; acetate, butyrate, lactate fermentation products; reported resistance to gentamicin, streptomycin, kanamycin	(76)
*Catenibacterium mitsuokai* subsp. *tridentinum* subsp. nov.	*Coprobacillaceae*	Feces from a healthy male in Italy	Not established	Anaerobic, non-motile, non-spore-forming Gram-positive bacillus; optimal growth at 37°C–40°C; optimal pH range 7.0–7.5; positive for maltose; negative for leucyl-glycine arylamidase; acetate fermentation products; reported resistance to streptomycin, kanamycin	(76)
*Clostridium amazonitimonense* sp. nov.	*Clostridiaceae*	Feces from a female in French Guyana	Not established	Anaerobic, motile, spore-forming, catalase-negative, oxidase-negative Gram-positive bacillus; 30-day preincubation in anaerobic blood culture bottle with rumen and sheep blood preceded cultivation of 5-mm diameter white colonies on Columbia agar with 5% sheep blood; optimal growth at 37°C in up to 0.5% NaCl; positive for ribose, mannose, alkaline phosphatase, D-fructose, D-glucose; negative for D-maltose, sucrose, D-lactose	(77)^*c*^
*Clostridium pacaense* sp. nov.	*Clostridiaceae*	Feces from a 3-month-old girl with severe malnutrition	Not established	Anaerobic, non-motile, spore-forming, catalase-negative, oxidase-negative Gram-variable bacillus; no *in vitro* cultivation parameters provided; positive for α-glucosidase; negative for indole, alkaline phosphatase, urease, ribose, mannose, mannitol, sucrose, β-galactosidase; susceptible to amoxicillin, ceftriaxone, cefepime, ertapenem, vancomycin, metronidazole	(78)^*c*^
*Coprococcus aceti* sp. nov.	*Lachnospiraceae*	Feces	Not established	Phenotypic characterization not provided; Gram reaction and atmospheric requirement predicted by genus assignment	(79)^*e*^
*Coprococcus immobilis* sp. nov.	*Lachnospiraceae*	Feces from a human with rheumatoid arthritis	Not established	Limited phenotypic characterization provided in reference 35; Gram reaction and atmospheric requirement predicted by genus assignment	(35)^*d*^
*Coprococcus intestinihominis* sp. nov.	*Lachnospiraceae*	Human feces	Not established	Phenotypic characterization not provided; Gram reaction and atmospheric requirement predicted by genus assignment	(50)^*c*^
*Coprococcus mobilis* sp. nov.	*Lachnospiraceae*	Feces from a human with rheumatoid arthritis	Not established	Limited phenotypic characterization provided in reference (35); Gram reaction and atmospheric requirement predicted by genus assignment	(35)^*d*^
*Coprococcus phoceensis* sp. nov.	*Lachnospiraceae*	Left colon cleansing specimen from patent with Crohn’s disease in France	Not established	Anaerobic, non-motile, catalase-negative, oxidase-negative Gram-variable bacillus; 3-mm diameter transparent, crater-shaped colonies on Columbia agar with 5% sheep blood; optimal growth at 37°C in < 0.5% NaCl; positive for potassium 5-ketogluconate, alkaline phosphatase, acid phosphatase, β-glucosidase, α-glucosidase, naphthol-AS-BI-phosphohydrolase; negative for D-glucose, D-lactose, D-xylose, valine arylamidase, C4 esterase, C8 esterase lipase, C14 lipase, urease, leucine arylamidase, salicin	(80)^*f*^
*Enterocloster hominis* sp. nov.	*Lachnospiraceae*	Human feces	Not established	Phenotypic characterization not provided; Gram reaction and atmospheric requirement predicted by genus assignment	(50)^*c*^
*Faecalibacillus hominis* sp. nov.	*Erysipelotrichaceae*	Feces from a human with rheumatoid arthritis	Not established	Limited phenotypic characterization provided in reference 35; Gram reaction and atmospheric requirement predicted by genus assignment	(35)^*d*^
*Finegoldia dalianensis* sp. nov.	*Peptoniphilaceae*	Purulent specimen from China	Female hospitalized with skin abscess	Anaerobic Gram-positive coccus; 1-mm diameter gray, smooth, circular colonies on Columbia agar with 5% sheep blood incubated at 37°C; positive for alanyl-phenylalanyl-proline arylamidase, β-galactopyranosidase indoxyl, 5-bromo-4-chloro-3-indoxyl-α-galactoside, 5-bromo-4-chloro-3-indoxyl-β-*N*-acetyl-glucosamide, hippurate; negative for leucine arylamidase, tyrosine arylamidase, urease	(81)^*d*^
*Haloimpatiens massiliensis* sp. nov.	*Clostridiaceae*	Feces from healthy female in Niger	Not established	Anaerobic, catalase-negative, oxidase-negative Gram-positive bacillus; 8-mm diameter gray, irregular colonies on Columbia agar with 5% sheep blood at 37°C; positive for α-fucosidase, acid phosphatase, α-chymotrypsin, alkaline phosphatase, C4 esterase, leucine arylamidase, amygdalin, inulin, esculin, salicin; negative for α-galactosidase, α-glucosidase, β-glucosidase, C8 esterase lipase, C14 lipase, valine arylamidase, cystine arylamidase, trypsin, L-arabinose, D-ribose	(82)^*c*^
*Lachnoanaerobaculum sanguinis* sp. nov.	*Lachnospiraceae*	Blood from a patient in Japan	Patient diagnosed with oral mucositis became bacteremic following chemotherapy for acute myeloid leukemia	Anaerobic, spore-forming Gram-positive bacillus; 0.23- to 0.75-mm diameter milky, circular (with undulate margin), glossy, non-hemolytic colonies on Columbia agar with 5% sheep blood; optimal growth at 35°C–37°C; positive for α-fucosidase, *N*-acetyl- β-glucosaminidase, indole, D-raffinose; weakly-positive for D-mannose; negative for urease, β-glucuronidase, pyroglutamic acid arylamidase, L-arabinose	(83)^*c*^
*Lachnospira acetigenes* sp. nov.	*Lachnospiraceae*	Feces from a human with rheumatoid arthritis	Not established	Limited phenotypic characterization provided in reference 35; Gram reaction and atmospheric requirement predicted by genus assignment	(35)^*d*^
*Lachnospira flagellata* sp. nov.	*Lachnospiraceae*	Feces from a human with rheumatoid arthritis	Not established	Limited phenotypic characterization provided in reference 35; Gram reaction and atmospheric requirement predicted by genus assignment	(35)^*d*^
*Lachnospira hominis* sp. nov.	*Lachnospiraceae*	Feces from a human with rheumatoid arthritis	Not established	Limited phenotypic characterization provided in reference 35; Gram reaction and atmospheric requirement predicted by genus assignment	(35)^*d*^
*Lachnospira rogosae* sp. nov.	*Lachnospiraceae*	Human feces	Not established	Phenotypic characterization not provided; Gram reaction and atmospheric requirement predicted by genus assignment	(50)^*c*^
*Lactonifactor hominis* sp. nov.	*Clostridiaceae*	Feces from a human with rheumatoid arthritis	Not established	Limited phenotypic characterization provided in referencce 35; Gram reaction and atmospheric requirement predicted by genus assignment	(35)^*d*^
*Megasphaera coli* sp. nov.	*Veillonellaceae*	Colonic material from man undergoing colonoscopic evaluation for iron deficiency anemia	Not established	Past designation of “*Colibacter massiliensis*" from clinical material never validly published; description followed two publications (84, 85), which noted Gram-positivity while noting assignment to *Veillonellaceae*; anaerobic, non-motile, non-spore-forming, catalase-negative, oxidase-negative Gram-positive diplococcus; 0.1-mm diameter circular, translucent colonies on Columbia agar with 5% sheep blood; positive for alkaline phosphatase, C4 esterase, C8 esterase lipase, leucine arylamidase, valine arylamidase, cystine arylamidase, α-chymotrypsin, β-galactosidase; negative for lipase, trypsin, α-galactosidase, α-fucosidase, glycerol, inulin, starch hydrolysis	(74)
*Methanobrevibacter intestini* sp. nov.	*Methanobacteriaceae*	Feces from a United States resident	Not established	Anaerobic, non-motile Gram-positive coccobacillus; optimal growth of colonies at 35-39°C and pH 7.0–7.5 on archaeal medium and at 37°C–39°C and pH 6.5 on modified MpT1 medium; growth dependent upon acetate; major lipid is archaeol; no other phenotypic data provided	(86)
*Peptoniphilus grossensis* sp. nov.	*Peptoniphilaceae*	Feces from a morbidly obese individual in France	Not established	Anaerobic, non-motile, spore-forming, catalase-negative, oxidase-negative Gram-positive coccus; 1-mm diameter colonies on Columbia and BHI agars; optimal growth at 37°C; positive for indole, D-mannose, arginine arylamidase, leucine arylamidase, tyrosine arylamidase; negative for nitrate reductase, urease, alanine arylamidase, serine arylamidase; reported resistance to ciprofloxacin; reported susceptibility to penicillin G, ceftriaxone, imipenem, fosfomycin, erythromycin, doxycycline, metronidazole	(87)^*c*^
*Peptoniphilus hominis* sp. nov.	*Peptoniphilaceae*	Feces from a human with rheumatoid arthritis	Not established	Limited phenotypic characterization provided in reference (35); Gram reaction and atmospheric requirement predicted by genus assignment	(35)^*d*^
*Peptoniphilus obesi* sp. nov.	*Peptoniphilaceae*	Feces from a morbidly obese individual in France	Not established	Anaerobic, non-motile, non-spore-forming, catalase-negative, oxidase-negative Gram-positive coccus; optimal growth at 37°C; positive for arginine arylamidase, leucine arylamidase; negative for urease, indole, D-mannose, alanine arylamidase, serine arylamidase, tyrosine arylamidase, histidine arylamidase	(88)^*c*^
*Peptoniphilus pacaensis* sp. nov.	*Peptoniphilaceae*	Vaginal discharge from a female in France with bacterial vaginosis	Not established	Anaerobic, non-motile, non-spore-forming, catalase-negative, oxidase-negative Gram-positive coccus; 1.0- to 1.5-mm diameter gray, translucent, circular colonies on Columbia agar; optimal growth at 37°C in less than 5% NaCl; positive for ribose, alkaline phosphatase; negative for indole, urease, β-galactosidase, *N*-acetyl-β-glucosaminidase, D-fructose	(89)^*c*^
*Pseudoramibacter faecis* sp. nov.	*Eubacteriaceae*	Feces from a volunteer in China	Not established	Anaerobic, non-motile, non-spore-forming, catalase-negative Gram-positive bacillus; 0.5- to 1.0-mm diameter white, opaque, oval, smooth, raised colonies on modified Gifu anaerobic medium (GAM) agar; optimal growth at 42°C in 0.0%–3.5% NaCl; positive for esculin, mannitol, C8 esterase lipase; negative for glucose, xylose, C4 esterase, alkaline phosphatase	(90)^*c*^
*Ruminococcoides intestinale* sp. nov.	*Oscillospiraceae*	Human feces	Not established	Phenotypic characterization not provided; Gram reaction and atmospheric requirement predicted by genus assignment	(50)^*c*^
*Ruminococcoides intestinihominis* sp. nov.	*Oscillospiraceae*	Human feces	Not established	Phenotypic characterization not provided; Gram reaction and atmospheric requirement predicted by genus assignment	(50)^*c*^
*Terrisporobacter muris* sp. nov.	*Peptostreptococcaceae*	Blood from patient in Republic of Korea	Sepsis secondary to colon perforation	Initial report of isolation from caecal content of specific pathogen-free mouse published in reference 69 and accepted in Validation List no. 221 (22); limited phenotypic characterization provided; Gram reaction and atmospheric requirement predicted by genus assignment	(91)
*Umbribacter vaginalis* gen. nov., sp. nov.	*Eggerthellaceae*	Isolates (2) from vaginal fluid of two patients with bacterial vaginosis in the United States	Not established	Anaerobic, non-motile, non-spore-forming, weakly catalase-positive, oxidase-negative Gram-variable coccobacillus; 0.3- to 0.4-mm diameter clear, punctiform, flat, shiny colonies on Brucella agar with 5% laked sheep blood; optimal growth at 30°C–37°C; resistant to colistin, susceptible to vancomycin (for identification purposes); positive for production of acetic, formic, fumic, pyruvic acids; negative for nitrate reduction, production of lactic and malonic acids	(92)
Gram-negative anaerobes					
*Akkermansia massiliensis* sp. nov.	*Akkermansiaceae*	Feces from an individual in France	Not established	Anaerobic, motile, non-spore-forming, oxidase-negative, catalase-positive Gram-negative bacillus; 0.5-mm diameter white, circular, convex, entire, non-hemolytic colonies on Columbia agar; optimal growth at 37°C in 0.5% NaCl; positive for maltose, ribose, cellobiose, D-fructose; negative for indole, urease, rhamnose, raffinose, xylose	(93)^*c*^
*Alistipes montrealensis* sp. nov.	*Rikenellaceae*	Feces from a female with colitis secondary to metastatic melanoma in Canada	Not established	Anaerobic, non-motile, non-spore-forming, catalase-positive, oxidase-negative Gram-negative bacillus; 0.2- to 0.3-mm diameter gray, circular, convex colonies on tryptic soy agar; optimal growth at 37°C in <0.5% NaCl; positive for indole, esculin hydrolysis, β-galactosidase, β-glucosidase, cellobiose, glucose, lactose, rhamnose; negative for nitrate reduction, gelatin digestion, mannitol; reported resistance to colistin, kanamycin, vancomycin; reported susceptibility to metronidazole	(94)^*g*^
*Anaerofustis butyriciformans* sp. nov.	*Eubacteriaceae*	Feces from a volunteer in China	Not established	Anaerobic, non-motile, non-spore-forming, catalase-negative Gram-negative bacillus; 0.3- to 0.5-mm diameter white, opaque, elliptical, smooth, raised colonies on modified GAM agar; optimal growth at 37°C in 0.0%–1.5% NaCl; positive for esculin, glucose, xylose, C4 esterase, C8 esterase lipase, alkaline phosphatase; negative for mannitol	(90)^*c*^
*Bacteroides celer* sp. nov.	*Bacteroidaceae*	Feces from a healthy male in Korea	Not established	Anaerobic, non-motile, non-spore-forming, catalase-negative, oxidase-negative, bile-tolerant Gram-negative bacillus; cream-colored, moist, entire, glistening, circular colonies on GAM agar; optimal growth at 37°C in 1% NaCl; positive for α-galactosidase, β-galactosidase, D-mannitol, salicin, D-melezitose, arabinose, D-melibiose; negative for glutamic acid decarboxylase	(95)^*g*^
*Bacteroides mucinivorans* sp. nov.	*Bacteroidaceae*	Feces from a healthy female in Korea	Not established	Anaerobic, non-motile, non-spore-forming, catalase-negative, oxidase-negative, bile-tolerant Gram-negative bacillus; cream-colored, moist, circular, glistening, entire colonies on GAM agar; optimal growth at 37°C in 1% NaCl; positive for glutamic acid decarboxylase; negative for α-galactosidase, β-galactosidase, D-mannitol, salicin, D-melezitose, arabinose, D-melibiose	(95)^*g*^
*Butyricimonas recta* sp. nov.	*Odoribacteraceae*	Feces from a human with rheumatoid arthritis	Not established	Limited phenotypic characterization provided in reference 35; Gram reaction and atmospheric requirement predicted by genus assignment	(35)^*d*^
*Dentiradicibacter hellwigii* gen. nov., sp. nov.	No current family assignment	Root canal of the upper left maxillary premolar from a female in Germany	Infected root canal	Anaerobic, motile, oxidase-negative, catalase-positive, slender Gram-negative bacillus; translucent colonies on yeast extract cysteine agar with 10% sheep blood; optimal growth at 36°C with *Capnocytophaga sputigena* co-culture; positive for glucose, maltose, lactose, fructose, mannitol; negative for citrate	(96)
*Desulfovibrio falkowii* sp. nov.	*Desulfovibrionaceae*	Feces from Japan	Female patient with Parkinson’s disease	Anaerobic, non-motile, non-spore-forming, catalase-positive Gram-negative bacillus; 0.2- to 0.4-mm diameter white, circular, slightly-raised, smooth colonies on GAM agar; optimal growth at 25°C–37°C in up to 3% NaCl; positive for hydrolysis of esculin ferric citrate; negative for urease, indole, nitrate reductase, leucine arylamidase, alkaline phosphatase, glycine arylamidase, D-glucose, D-mannitol, D-lactose, D-sucrose; major cellular fatty acids iso-C_15:0_, C_18:1_ω9c	(97)
*Eisenbergiella longa* sp. nov.	*Lachnospiraceae*	Feces from a human with rheumatoid arthritis	Not established	Limited phenotypic characterization provided in reference 35; Gram reaction and atmospheric requirement predicted by genus assignment	(35)^*d*^
*Faecalibacterium intestinale* sp. nov.	*Oscillospiraceae*	Human feces	Not established	Phenotypic characterization not provided; Gram reaction and atmospheric requirement predicted by genus assignment	(50)^*c*^
*Faecalibacterium langellae* sp. nov.	*Oscillospiraceae*	Feces from healthy adult	Not established	Non-validly-published “*Faecalibacterium langellae*” homonym found in reference 98; anaerobic, catalase-negative Gram-negative bacillus; cultivated in BHI-supplemented broth at 30°C–42°C; inhibited by ≥ 1% NaCl; negative for gluconate, alkaline phosphatase, C4 esterase, C8 esterase lipase, α-galactosidase, β-glucuronidase, α-glucosidase; weakly-positive for sucrose, mannose	(99)
*Faecalibacterium wellingii* sp. nov.	*Oscillospiraceae*	Feces from a healthy volunteer in the Netherlands	Not established	Anaerobic Gram-variable bacillus with tapered ends; white/grayish, circular, umbonate colonies on yeast, casitone, short-chain fatty acids glucose (YCFAG) agar; growth at 37°C–42°C in up to 0.1% NaCl; consumes acetate and produces butyrate in YCFAG medium; biofilm production	(98)^*e*^
*Flavonifractor hominis* sp. nov.	*Oscillospiraceae*	Human feces	Not established	Phenotypic characterization not provided; Gram reaction and atmospheric requirement predicted by genus assignment	(50)^*c*^
*Leptotrichia alba* sp. nov.	*Leptotrichiaceae*	Dental biofilm from a healthy volunteer aged 2–45 years in China	Not established	Anaerobic, non-motile, non-spore-forming, oxidase-negative, catalase-negative Gram-negative bacillus; 5-mm diameter round, rough colonies with slightly-raised white center on BHI agar at 30°C–42°C in up to 1% NaCl; positive for alkaline phosphatase, naphthol-AS-BI-phosphatase, phenylalanine arylamidase, glutamate decarboxylase; negative for β-galactosidase-6-phosphate, pyroglutamic arylamidase, D-sorbitol	(100)^*b*^
*Leptotrichia mesophila* sp. nov.	*Leptotrichiaceae*	Dental biofilm from a healthy volunteer aged 20–45 years in China	Not established	Anaerobic, non-motile, non-spore-forming, oxidase-negative, catalase-negative Gram-negative bacillus; 1-mm diameter off-white, rough colonies on BHI agar at 25°C–42°C in up to 1% NaCl; positive for alkaline phosphatase, naphthol-AS-BI-phosphatase, pyroglutamic arylamidase, D-sorbitol; negative for β-galactosidase-6-phosphate, phenylalanine arylamidase, glutamate decarboxylase	(100)^*b*^
*Leptotrichia rugosa* sp. nov.	*Leptotrichiaceae*	Dental biofilm from a healthy volunteer aged 20–45 years in China	Not established	Anaerobic, non-motile, non-spore-forming, oxidase-negative, catalase-negative Gram-negative bacillus; 3- to 5-mm diameter off-white, rough colonies on BHI at 30°C–42°C in up to 1% NaCl; positive for β-galactosidase-6-phosphate, phenylalanine arylamidase, pyroglutamic arylamidase; negative for alkaline phosphatase, naphthol-AS-BI-phosphatase, glutamate decarboxylase, D-sorbitol	(100)^*b*^
*Mediterraneibacter flintii* sp. nov.	*Lachnospiraceae*	Feces from Japan	Female patient with Parkinson’s disease	Anaerobic, non-motile, non-spore-forming, catalase-positive Gram-negative bacillus; 0.2- to 0.4-mm diameter cream/white, circular or irregular, flat colonies with undulated margins on GAM agar; optimal growth at 32°C–42°C in up to 2% NaCl; positive for sucrose, maltose, salicin, cellobiose, mannose, α-glucosidase; negative for gelatin hydrolysis, esculin hydrolysis, mannitol, lactose, xylose, arabinose, glycerol, arginine dihydrolase, L-glutamine	(97)
*Owariibacterium komagatae* gen. nov., sp. nov.	*Oscillospiraceae*	Feces from Japan	Female patient with Parkinson’s disease	Anaerobic, non-motile, non-spore-forming, catalase-negative Gram-negative bacillus; 0.3- to 0.6-mm diameter white, circular, slightly-raised colonies with smooth margins on GAM agar; optimal growth at 32°C–42°C in up to 1% NaCl; positive for lactose; negative for sucrose, maltose, salicin, esculin hydrolysis, glycerol, mannose, melezitose, raffinose, rhamnose	(97)
*Parabacteroides propionicigenes* sp. nov.	*Tannerellaceae*	Feces from a human with rheumatoid arthritis	Not established	Limited phenotypic characterization provided in reference 35; Gram reaction and atmospheric requirement predicted by genus assignment	(35)^*d*^
*Parabacteroides timonensis* sp. nov.	*Tannerellaceae*	Feces from healthy male in Congo	Not established	Anaerobic, motile, non-spore-forming, catalase-positive, oxidase-negative Gram-negative bacillus; 0.9- to 1.0-mm diameter smooth colonies on Columbia agar with 5% sheep blood; optimal growth at 37°C in 5% NaCl; positive for D-ribose, L-arabinose, β-galactosidase, D-mannose, D-mannitol; negative for indole, urease; increased minimum inhibitory concentration values for metronidazole, amikacin	(101)^*c*^
*Phocaeicola acetigenes* sp. nov.	*Bacteroidaceae*	Feces from a healthy female in Korea	Not established	Anaerobic, non-motile, non-spore-forming, catalase-negative, oxidase-negative Gram-negative bacillus; round, translucent, convex, entire colonies on GAM; optimal growth at 37°C in non-salinic conditions; positive for D-glucose, D-xylose, α-galactosidase, α-glucosidase, α-fucosidase; negative for L-arabinose, esculin ferric citrate, D-cellobiose, D-rhamnose, β-glucosidase, arginine arylamidase, leucine arylamidase, glycine arylamidase, serine arylamidase	(102)^*g*^
*Porphyromonas miyakawae* sp. nov.	*Porphyromonadaceae*	Feces from Japan	Female patient with Parkinson’s disease	Anaerobic, non-motile, non-spore-forming, catalase-positive Gram-negative bacillus; 0.2- to 0.4-mm diameter opaque, circular, convex colonies with smooth margins that become black-pigmented over time on Columbia agar with 5% horse blood; optimal growth at 32°C–42°C; positive for L-tryptophan hydrolysis, indole, alkaline phosphatase, acid phosphatase, naphthol-AS-BI-phosphohydrolase; negative for C8 esterase lipase, C14 lipase, arginine arylamidase, α-fucosidase, *N*-acetyl-β-glucosaminidase	(97)
*Pseudoflavonifractor intestinihominis* sp. nov.	*Oscillospiraceae*	Human feces	Not established	Phenotypic characterization not provided; Gram reaction and atmospheric requirement predicted by genus assignment	(50)^*c*^
*Pyramidobacter arthritidis* sp. nov.	*Dethiosulfovibrionaceae*	Feces from a human with rheumatoid arthritis	Not established	Limited phenotypic characterization provided in reference 35; Gram reaction and atmospheric requirement predicted by genus assignment	(35)^*d*^
*Ruthenibacterium intestinale* sp. nov.	*Oscillospiraceae*	Human feces	Not established	Phenotypic characterization not provided; Gram reaction and atmospheric requirement predicted by genus assignment	(50)^*c*^
*Veillonella orientalis* sp. nov.	*Veillonellaceae*	Saliva of a healthy boy from Thailand	Not established	Anaerobic, non-motile, non-spore-forming, catalase-negative Gram-negative coccus; 0.5- to 2-mm diameter gray/white, circular, smooth, opaque, non-hemolytic colonies on BHI blood agar at 37°C; positive for arginine dihydrolase, nitrate reductase, alkaline phosphatase, pyroglutamic acid arylamidase, C4 esterase, C8 esterase lipase, acid phosphatase; reported resistance to vancomycin; reported susceptibility to colistin, kanamycin, metronidazole	(103)^*d*^
*Waltera hominis* sp. nov.	*Lachnospiraceae*	Human feces	Not established	Phenotypic characterization not provided; Gram reaction and atmospheric requirement predicted by genus assignment	(50)^*c*^

^*a*^ Number of isolates from clinical source is 1, except where shown in parentheses.

^*b*^ Validly published by inclusion in Validation List no. 224 (55).

^*c*^ Validly published by inclusion in Validation List no. 225 (54).

^*d*^ Validly published by inclusion in Validation List no. 223 (53).

^*e*^ Validly published by inclusion in Validation List no. 221 (22).

^*f*^ Validly published by inclusion in Validation List no. 222 (56).

^*g*^ Validly published by inclusion in Validation List no. 226 (71).

**TABLE 4:** Novel, validly published, spiral-shaped bacterial species recovered from human clinical material reported from January 2025 through December 2025

Scientific name	Family	Source^*a*^	Clinical relevance	Growth characteristics	Reference(s)
*Fluviispira vulneris* sp. nov.	*Silvanigrellaceae*	Wound from a patient in China	Wound tissue and secretion from a patient with bleeding from right leg injury; elevated innate immunity metrics	Aerobic, non-motile, oxidase-negative, catalase-positive Gram-negative pleomorphic bacillus (some spirals observed); red, round, smooth, convex colonies on Reasoner’s 2A agar; optimal growth at 28°C–32°C in up to 1.5% NaCl; positive for leucine arylamidase, alkaline phosphatase, acid phosphatase; negative for gelatin hydrolysis, β-galactosidase, glucose	(104)^*b*^

^*a*^ Number of isolates from clinical source is 1, except where shown in parentheses.

^*b*^ Validly published by inclusion in Validation List no. 226 (71).

**TABLE 5:** Novel, validly published bacterial species not characterized by Gram reaction that were recovered from human clinical material reported from January 2025 through December 2025

Scientific name	Family	Source^*a*^	Clinical relevance	Growth characteristics	Reference(s)
Anaerobic bacteria not characterized by Gram reaction					
*Bovifimicola ammoniilytica* gen. nov., sp. nov.	*Lachnospiraceae*	Feces	Not established	Phenotypic characterization not provided	(79)^*b*^
*Brotomerdimonas butyrica* gen. nov., sp. nov.	No current family assignment	Feces	Not established	Phenotypic characterization not provided	(79)^*b*^
*Porcipelethomonas ammoniilytica* gen. nov., sp. nov.	*Oscillospiraceae*	Feces	Not established	Phenotypic characterization not provided	(79)^*b*^
Other bacteria not characterized by Gram reaction					
*Jirenia arthritidis* gen. nov., sp. nov.	*Oscillospiraceae*	Feces from a human with rheumatoid arthritis	Not established	Limited phenotypic characterization provided in (35)	(35)^*c*^
*Hominiventricola aquisgranensis* sp. nov.	*Lachnospiraceae*	Human feces	Not established	Phenotypic characterization not provided	(50)^*d*^
*Laedolimicola intestinihominis* sp. nov.	*Lachnospiraceae*	Human feces	Not established	Phenotypic characterization not provided	(50)^*d*^
*Markusia hominis* gen. nov., sp. nov	*Oscillospiraceae*	Feces from a human with rheumatoid arthritis	Not established	Limited phenotypic characterization provided in reference 35	(35)^*c*^
*Naizhengia acetigignens* gen. nov., sp. nov.	*Lachnospiraceae*	Feces from a human with rheumatoid arthritis	Not established	Limited phenotypic characterization provided in reference 35	(35)^*c*^
*Sanxizhangella immobilis* gen. nov., sp. nov.	*Lachnospiraceae*	Feces from a human with rheumatoid arthritis	Not established	Limited phenotypic characterization provided in reference 35	(35)^*c*^
*Solibaculum intestinale* sp. nov.	*Oscillospiraceae*	Human feces	Not established	Phenotypic characterization not provided; other species designation of the *Solibaculum* genus documented to be Gram-variable (105)	(50)^*d*^
*Ventrimonas faecis* gen. nov., sp. nov.	*Lachnospiraceae*	Human feces	Not established	Phenotypic characterization not provided	(50)^*d*^
*Yonghella fusiformis* gen. nov., sp. nov.	*Lachnospiraceae*	feces from a human with rheumatoid arthritis	Not established	Limited phenotypic characterization provided in reference 35	(35)^*c*^
*Zhonglingia intestinalis* gen. nov., sp. nov.	*Erysipelotrichaceae*	Feces from a human with rheumatoid arthritis	Not established	Limited phenotypic characterization provided in reference 35	(35)^*c*^

^*a*^ Number of isolates from clinical source is 1, except where shown in parentheses.

^*b*^ Validly published by inclusion in Validation List no. 221 (22).

^*c*^ Validly published by inclusion in Validation List no. 223 (53).

^*d*^ Validly published by inclusion in Validation List no. 225 (54).

**TABLE 6:** Revised bacterial taxa relative to human clinical material accepted from January 2025 through December 2025

Earlier validly published name	2025 Validly published name	Other information	Reference(s)
Gram-positive cocci			
*Streptococcus chosunensis*	*Streptococcus mitis*	Descriptions of the *Streptococcus chosunense* taxon from maxillary cyst material found in references “^5^, ^106^” and included as *S. chosunensis* (107); *S. chosunensis* is a synonym of *S. mitis*	(40)
*Streptococcus downii*	*Streptococcus oralis* subsp. *dentisani*	Descriptions of the *S. downii* taxon from oral cavity material found in references “^34^, ^108^”; *S. downii* is a synonym of *S. oralis* subsp. *dentisani*	(40)
*Streptococcus gwangjuensis*	*Streptococcus mitis*	Descriptions of the *Streptococcus gwangjuense* taxon from human pericoronitis found in references “^5^, ^109^” and included as *S. gwangjuensis* (107); *S. chosunensis* is a synonym of *S. mitis*	(40)
*Streptococcus hohhotensis*	*Streptococcus mitis*	Descriptions of the *S. hohhotensis* taxon from breast milk found in references “^5^, ^110^”; *S. hohhotensis* is a synonym of *S. mitis*	(40)
*Streptococcus humanilactis*	*Streptococcus mitis*	Descriptions of the *S. humanilactis* taxon from breast milk found in references 5, 12 and included in reference 107; *S. humanilactis* is a synonym of *S. mitis*	(40)
*Streptococcus nakanonensis*	*Streptococcus thalassemiae*	Refer to Table 1 for description of the *S. nakanonensis* taxon; *S. nakanonensis* is a synonym of *S. thalassemiae*	(40)
*Streptococcus parapneumoniae*	*Streptococcus thalassemiae*	Refer to Table 1 for description of the *S. parapneumoniae* taxon; *S. parapneumoniae* is a synonym of *S. thalassemiae*	(40)
*Streptococcus toyakuensis*	*Streptococcus mitis*	Descriptions of the *S. toyakuensis* taxon from blood found in references “^7^, ^111^” and included in reference 112; *S. toyakuensis* is a synonym of *S. mitis*	(40)
Gram-positive bacilli			
*Pseudonocardia carboxydivorans*	*Pseudonocardia alni* subsp. *carboxydivorans* comb. nov.	Initial description of the *P. carboxydivorans* taxon from soil found in reference 113; report of isolation from cerebrospinal fluid found in reference 114	(115)
*Virgibacillus massiliensis*	*Virgibacillus salexigens*	Descriptions of the *V. massiliensis* taxon from feces found in references “^6^, ^116^” and included in reference 117; *V. massiliensis* is a synonym of *V. salexigens*	(118)
*Zimmermannella alba*	*Pseudoclavibacter albus* comb. nov.	Initial description of the *Z. alba* taxon from human urine found in reference 119	(120)^*a*^
Gram-negative coccus			
*Roseomonas aerofrigidensis*	*Teichococcus aerofrigidensis* comb. nov.	Initial description of the *R. aerofrigidensis* taxon from an environmental source found in reference 121; report of isolation in the context of infectious endocarditis found in reference 122	(21)^*b*^
Gram-positive anaerobes			
*Bifidobacterium faecale*	*Bifidobacterium adolescentis*	Descriptions of the *B. faecale* taxon from feces found in references “^31^, ^123^”; *B. faecale* is a synonym of *B. adolescentis*	(124)
*Blautia coccoides*	*Blautia producta*	Initial valid publication and inclusion of this organism was the taxon *Peptostreptococcus productus* (125, 126); interim designations of *Clostridium coccoides* (126, 127), *Ruminococcus productus* (128, 129), and the reclassification of six anaerobic Gram-positive bacilli into the *B. coccoides* taxon (130) have been noted; isolation of this bacterium in the context of Alzheimer’s disease found in reference 131; *B. coccoides* is a synonym of *B. producta*	(132)
Gram-negative anaerobes			
*Anaeroglobus geminatus*	*Megasphaera geminata* comb. nov.	Initial description of the *A. geminatus* taxon from post-operative gastrointestinal fluid collection found in reference 133	(74)
*Allisonella histaminiformans*	*Dialister histaminiformans* comb. nov.	Initial description of the *A. histaminiformans* taxon from bovine and equine sources found in reference 134 and included in reference 135; report of taxon significance in humans found in reference 136	(74)
*Selenobaculum gibii*	*Anaerosinus gibii* comb. nov.	Descriptions of the *Selenobaculum gbiensis* taxon from feces of a Crohn’s disease patient found in references “^5^, ^137^” and included in reference 138	(74)
*Suonthocola fibrivorans*	*Diplocloster agilis*	*S. fibrivorans* initially characterized only by genotypic means in reference 79; Gram reaction and oxygen requirement assumed by new genus assignment (7, 139); *S. fibrivorans* is a synonym of *D. agilis*	(140)

^*a*^ Validly published by inclusion in Validation List no. 223 (101).

^*b*^ Validly published by inclusion in Validation List no. 221 (22).

**TABLE 7:** Update on clinical relevance for selected novel taxa described in *Journal of Clinical Microbiology* (*JCM*) taxonomy compendia published in 2017, 2021, 2023, and 2024

Taxon	*JCM* compendium	Human source(s) initially reported	Example(s) of clinical update	Reference(s)
*Faucicola mancuniensis*	2017 (31)	Tonsils of healthy adult female	Isolation via pharyngeal swab surveillance of (healthy) close contacts of epidemic cerebrospinal meningitis patients in China	(141)
*Enterobacter chengduensis*	2021 (33)	Blood	Four carbapenem-resistant strains isolated from intensive care unit or gastroenterology patients in China	(142)
*Enterobacter chuandaensis*	2021 (33)	Blood	Carbapenem-resistant isolate from perianal swab of intensive care unit a patient in China	(143)
*Pseudomonas juntendi*	2021 (33)	Sputum, urine	Urine isolate from China harboring two metallo-β-lactamase determinants	(144)
*Pandoraea pneumonica*	2023 (34)	Not specified (cystic fibrosis patient from United States)	Cluster of bloodstream infections in China; 17% mortality rate	(145)
*Corynebacterium hesseae*	2024 (6)	Urine, skin, vagina/rectum, sebaceous cyst, nasal mucosa	Bloodstream infection in Brazilian patient	(146)
*Gulosibacter massiliensis*	2024 (6)	Blood	Traumatic wound infection; uneventful clinical course following antimicrobial therapy	(147)

A total of 50 taxa itemized in Tables 1 to 3 and 5 comes from four publications (35, 50, 69, 79) encompassing largely genotypic findings from mammalian microbiome investigations. Gram morphology data were not available; classification of 37 taxa within Tables 1 to 3 was therefore made on the basis of historic knowledge of Gram reaction and atmospheric requirement of their assigned genus. Historically, these microbiome investigations involved the initial propagation of organisms in an anaerobic environment; as such, past *Journal of Clinical Microbiology* compendia (5–7) have categorized novel genus designations for which a Gram morphology was not reported (i.e., genera which did not have a previously-published species designation for comparison) as “Anaerobic bacterium not characterized by Gram reaction”. Recent publications from Huang et al. (35) and Hitch et al. (50) report organisms such as *Enterococcus* spp., *Streptococcus* spp., and *Enterobacter* spp. being derived from these culture systems. As such, the final stratification of Table 5 now reads “Other bacteria not characterized by Gram reaction” to account for the possibility of facultative bacteria. Two of these genotypically described taxa were initially reported by Afrizal et al. (69) as being derived from a murine host. Reports from Liu et al. (70) and Kang et al. (91) have chronicled these bacteria in the context of clinically significant infections in humans.

### Novel taxa

Among the novel Gram-positive cocci, species within the genera *Enterococcus*, *Macrococcus*, and *Streptococcus* are represented (Table 1). Most of the new species do not yet have established clinical relevance, and in many cases, represent members of the human microbiota.

Mašlaňová et al. characterized six novel *Macrococcus* isolates from food-producing animals and human clinical samples (37). These isolates were similar to other *Macrococcus* spp. in that they were catalase-positive, coagulase-negative, oxidase-positive Gram-positive cocci that grew in clusters and tetrads. Uniquely, these particular isolates were capable of growth in 1°C tryptone soy broth and hence were given the species name *psychrotolerans*. Three of the isolates were found to harbor large MecB-encoding plasmids that were easily transferred to *Staphylococcus aureus* by conjugation experiments. The combination of frequent contamination of foods, especially dairy products, the ability to tolerate refrigeration temperatures, and the presence of MecB-mediated β-lactam resistance raises concerns that this opportunistic organism has the potential to pose a risk to human health by way of foodborne illness (37).

Fourteen novel *Streptococcus* spp. have been characterized within the last year. Among these, three species were recovered from the breast milk of healthy women in China, namely *Streptococcus fermentans* sp. nov., *Streptococcus jiangnanensis* sp. nov., and *Streptococcus wuxiensis* sp. nov. (39). Through genomic annotation, the authors determined that the three strains possessed extensive capabilities for carbohydrate metabolism, particularly human milk oligosaccharide utilization (39), similar to other streptococci found in human milk microbiota (148). Human milk microbiota significantly impacts infant gut microbiome composition, leading to a variety of physiological benefits (149).

Five novel *Streptococcus* spp. were recovered from patients with significant infections. Three patients from different geographic regions in Asia had secondary bacteremia with three different novel streptococci. *Streptococcus taonis* sp. nov. was recovered from the blood culture of a pediatric patient hospitalized in a hospital in Taiwan with fever, croup, and suspected bacterial infection (43). The authors note that the single blood culture was obtained from a blood vessel on the dorsal hand of the patient, and no other cultures were positive. That said, the patient was treated with broad-spectrum antimicrobial therapy. This α-hemolytic isolate was most closely related to *Streptococcus vulneris* sp. nov. (46).

A 21-month-old child from Taoyuan City in China was hospitalized with croup and radiological findings of bronchopneumonia (44). Blood cultures were positive for an α-hemolytic *Streptococcus* spp. that was originally identified as *Streptococcus parasanguinis*. Subsequent whole genome sequencing revealed average nucleotide identity values (ANI) with the closest related streptococci of 94.1% or less (cutoff value 95%). Digital DNA-DNA hybridization (dDDH) values ranged from 22.5% to 55.6% (cutoff value 70%) compared *with S. parasanguinis* and related species, indicating that this isolate was a new species. Of note, this effectively published *Streptococcus taoyuanensis* sp. nov. taxon was resistant to clindamycin and erythromycin but susceptible to cefotaxime and vancomycin (44).

In Japan, a 60-year-old man with a complex medical history, including diabetes mellitus, was admitted for general debilitation and disorientation. On examination, he was febrile and tachycardic, with tenderness at the left costovertebral angle. Laboratory studies demonstrated leukocytosis and an elevated C-reactive protein (2.6 mg/dL). Computed tomography confirmed acute pyelonephritis of the left kidney. A blood culture was positive for an α-hemolytic *Streptococcus* spp. that was bile-soluble and optochin-susceptible, yet was Quellung-negative and negative for *lytA* (encoding pneumococcal autolysin LytA), indicating that the organism was likely not *Streptococcus pneumoniae* (42). The patient was treated with ampicillin-sulbactam and recovered. The isolate was resistant to levofloxacin, erythromycin, and clindamycin; intermediate to penicillin; and susceptible to ceftriaxone, meropenem, vancomycin, and linezolid. Phylogenetic studies revealed that the recovered organism, while closely related to *S. pneumoniae* and *Streptococcus pseudopneumoniae*, satisfied criteria for classification as a new species, which was designated *Streptococcus parapneumoniae* sp. nov. (42). This new species also lacked capsular material and other virulence factors associated with *S. pneumoniae*. As per Table 6, *S. parapneumoniae* was later determined to be a heterotypic synonym of *Streptococcus thalassemiae*.

Interestingly, another quinolone-resistant α-hemolytic *Streptococcus* spp. was isolated from sputum of an elderly man who presented with pneumonia in Nakano, Japan (41). The organism was initially identified as *S. pneumoniae* by an automated instrument and demonstrated bile solubility. Because quinolone resistance among *S. pneumoniae* is unusual in Japan, the isolate was further characterized by whole genome sequencing and expanded phenotypic testing. The organism had an ANI by BLAST of 93.69% and a dDDH value of 56.0% compared with the closest related species, *S. pseudopneumoniae*. Therefore, the authors determined the isolate to be a new species, which they named *Streptococcus nakanonensis* sp. nov. (41). As per Table 2, *S. nakanonensis* was later determined to be a heterotypic synonym of *S. thalassemiae*.

Finally, in a survey of patients with diabetic foot ulcers, a unique *Streptococcus* spp. was recovered from the ulcer of one patient. Complete genotypic characterization resulted in the designation *S. vulneris* sp. nov. Additional information on patient status was not provided (46).

Oftentimes, organisms recovered from human specimens fail to identify using standard phenotypic and proteomic methods and are therefore referred to reference laboratories for more complete characterization. This was the case with most of the novel species of aerobic Gram-positive bacilli listed in Table 1. Although two novel species, *Corynebacterium haematomassiliense* sp. nov. and *Corynebacterium mayonis* sp. nov.*,* were recovered from the blood of two separate patients, their contributions to clinical disease in the patients were not elucidated in the two studies (25, 26). In contrast, *Scrofimicrobium appendicitidis* sp. nov., a facultative Gram-positive bacillus in the family *Actinomycetaceae,* was recovered from a patient with ruptured appendicitis in Hong Kong (51). The 60-year-old patient was admitted with fever and right lower quadrant abdominal pain. A computed tomography scan of the abdomen and pelvis showed acute appendicitis complicated by abscess formation. A peritoneal swab obtained at the time of laparoscopic appendectomy grew *Escherichia coli*, *Bacteroides thetaiotaomicron*, and an unknown Gram-positive bacillus, later determined to be a novel species by whole genome sequencing (most closely related to *Scrofimicrobium canadense*). The patient recovered after surgery and treatment with ertapenem and later amoxicillin-clavulanate (51).

The inception of order *Enterobacterales* took place in 2016 (150) with the creation of seven families (i.e., six novel families in addition to retained family designation *Enterobacteriaceae*). An eighth family, *Gallaecimonadaceae*, was added in 2023 (151) and included in 2024 (152). Calendar year 2025 saw more than one dozen novel taxa added or included in this order (Table 2). To family *Erwiniaceae* was included *Mixta hanseatica* sp. nov. by way of Validation List no. 226 (71). In 2023, Both et al. (60) reported a Gram-negative bacillus being isolated from blood culture of an elderly patient with multiple skin abrasions and cellulitis of the left hip. Innate immunity metrics at the time of encounter included a C-reactive protein level of 281 mg/L, which decreased to 164 mg/L upon initiation of ampicillin-sulbactam therapy. Matrix-assisted laser desorption ionization–time of flight mass spectrometry (MALDI-TOF MS) analysis of the isolate rendered a genus-level *Erwinia* spp. identification with an identification score above 2.0. Initial 16S rRNA gene sequencing yielded similarity matches with *Pantoea alhagi*, *Mixta theicola*, and *Erwinia amylovora*. Whole genome sequencing subsequently revealed 83.2% ANI with closest relative *M. theicola*. Therapy was discontinued on day three due to decreases in C-reactive protein values, and the patient was transferred to a rehabilitation department. Later increases in C-reactive protein determinations in that unit resulted in an 8-day regimen of ceftriaxone (without additional blood culture collection). The cellulitis was noted to resolve.

A total of six novel taxa were included in family *Morganellaceae* via Validation Lists (22, 55, 71) in 2025. *Proteus appendicitidis* sp. nov. (61) varies from other *Proteus* spp. by its saline tolerance range (0-6%) and optimal growth temperature (30°C). This swarming isolate (swarming phenotype lost upon culture in anaerobic conditions) was recovered from appendiceal pus collected from an afebrile a patient in China with acute appendicitis and localized peritonitis. Leukocytosis (with greater than 83% neutrophils) and an elevated C-reactive protein level were noted upon admission. Initial commercial biochemical system identification of the isolate revealed *Proteus hauseri*. Phylogenetic analysis suggested *Proteus columbae* and *Proteus alimentorum* as closest relatives. The isolate was resistant to ampicillin, nitrofurantoin, cefaclor, and cefuroxime.

Four additional primary publications describe five novel *Providencia* spp. taxa recovered from patients in China. *Providencia xianensis* sp. nov. (63) was isolated from two patients in two distinct geographic locations and further distinguished on a temporal basis. One patient presented with septic shock and proteinuria, while a second was diagnosed with intra-abdominal infection (with the isolate recovered from ascites fluid). Both isolates were identified as *Providencia rettgeri* via MALDI-TOF MS and generated a characteristic susceptibility profile for *Morganellaceae* that included resistance to colistin, polymyxin B, tetracycline, and tigecycline. The isolates were further resistant to ciprofloxacin, imipenem, and trimethoprim-sulfamethoxazole, while susceptible to aztreonam and amikacin. Varying susceptibility was noted for carbapenem agents and cefepime. *bla*_NDM-1_ and *bla*_DHA-1_ determinants were noted among the two isolates. *P. xianensis* is positive for urea hydrolysis, citrate utilization, and deaminase activity, while negative for decarboxylase activity.

The remaining four novel *Providencia* spp. taxa were published by Dong and colleagues and recovered from a variety of specimen sources. An additional species misidentified as *P. rettgeri* by MALDI-TOF MS, namely *Providencia hangzhouensis* sp. nov. (62), was isolated from a patient with urinary tract infection in 2021, with the isolate revealing broad resistance to cephem agents. Decarboxylase, dihydrolase, urease, citrate, and deaminase reactions were consistent with many *Providencia* spp. ANI values of less than 92% were demonstrated for *P. rettgeri* and ten additional *Providencia* spp. when compared with the novel taxon. Subsequent population genomic surveillance of archived *P. rettgeri* and *Providencia huaxiensis* revealed the emergence of *P. hangzhouensis* in several nations, including the United States, China, and Nigeria. Nearly 85% of nearly 100 characterized genomes possessed extended-spectrum β-lactamase (ESBL) or carbapenem resistance determinants; nearly one-half of genomes carried both. Predominant ESBL and carbapenem resistance genes included *bla*_OXA-10_ and *bla*_NDM-27_, respectively.

A detailed analysis of 545 *Providencia* spp. genomes (65) resulted in the characterization of *Providencia zhijiangensis* sp. nov. The basis for this study was the alleged isolation of *Providencia alcalifaciens* (via MALDI-TOF MS) from bile of a patient who was admitted for acute suppurative cholangitis and septic shock. The patient was tachycardic and tachypneic upon admission, with a peripheral leukocyte count of 27 × 10^9^/mL (90% neutrophils) and a C-reactive protein level of 261 mg/L. No other culture findings were provided by the authors. Impaired consciousness was also noted. Again, decarboxylase, citrate, and deaminase reactions for this actual *P. zhijiangensis* isolate were consistent with those for many *Providencia* spp.; however, urea hydrolysis was not present. A total of 23 monophyletic lineages, including *P. zhijiangensis* (five genomes assessed) and at least seven yet-to-be-named taxa, were revealed in the overall genomic analysis. Diverse resistance profiles, including a propensity for plasmid carriage, were reported for approximately 80% of these lineages.

Dong et al. (64) also introduced the taxa *Providencia xihuensis* sp. nov. and *Providencia zhejiangensis* sp. nov., as isolated from blood and feces, respectively. The latter taxon was derived from a female with chronic myeloid leukemia in blast crisis who was admitted for assessment of a 4-day history of fever. At admission, leukocytosis with left shift and elevated C-reactive protein values were noted. No other microbiologic culture data were provided, other than the stool culture isolate that was originally identified via MALDI-TOF MS as *Providencia stuartii*. The patient did not respond clinically to a q12h regimen of aztreonam, and the clinical course progressed to septic shock, viral encephalitis, and palliative care. During admission of a 60-year-old male for upper abdominal pain, *P. xihuensis* was isolated from blood culture and initially identified by MALDI-TOF MS as *P. rettgeri*. Laparoscopic pancreaticoduodenectomy, two exploratory laparotomies, and digital subtraction angiography-guided embolizations due to abdominal hemorrhage complicated the clinical course. Patient status continued to deteriorate despite administration of cefoperazone-sulbactam and imipenem-cilastatin. The authors proposed the aforementioned two species designations to satisfy the previously unnamed status of taxon 4 and taxon 7 in a previous publication (65). Phenotypic antimicrobial susceptibility testing of the *P. zhejiangensis* isolate, implementing guidelines promulgated by CLSI and the European Committee on Antimicrobial Susceptibility Testing (EUCAST), revealed resistance to all β-lactam agents tested (mediated, in part, by a *bla*_NDM-1_ determinant) with the exception of aztreonam. In addition, fluoroquinolone and trimethoprim-sulfamethoxazole resistance was noted; variable *in vitro* aminoglycoside susceptibility was demonstrated. In contrast, the *P. xihuensis* isolate recovered from blood culture exhibited susceptibility to most β-lactam agents outside of ampicillin. However, through retrospective archival genome analysis, Dong et al. described a subclade of *P. xihuensis* with multiple acquired resistance genes that suggested horizontal gene transfer and means of rapid adaptation within the species.

To family *Enterobacteriaceae*, six taxa have been included either by primary publication or two Validation Lists (22, 54). He et al. (58) effectively published two *Enterobacter* spp. from blood culture isolation in separate febrile hospitalized patients, namely *Enterobacter chinensis* sp. nov. and *Enterobacter rongchengensis* sp. nov. No other pathogens were detected from either patient in blood culture analysis; data regarding number of blood culture bottles or sets were not divulged. Immunocompromised status was present in both patients, manifested as (i) neutropenia secondary to cancer chemotherapy or (ii) immunosuppressant administration following renal transplantation. Isolates were not matched to specific form of immunocompromise in the publication. Both isolates were identified as *Enterobacter cloacae* complex by a commercial biochemical system (despite the *E. chinensis* isolate not exhibiting arginine dihydrolase activity) and as *Enterobacter bugandensis* (32) via MALDI-TOF MS. Organisms demonstrating closest ANI values to the *E. chinensis* and *E. rongchengensis* isolates were *Enterobacter asburiae* and *Enterobacter chengduensis* (33), respectively. While neither patient developed septic shock, genetic characterization of the isolates revealed a capsular polysaccharide determinant within *E. chinensis* and those for hemolysin and shiga-like toxin in *E. rongchengensis*. Phenotypic antimicrobial susceptibility profiles for the two isolates resembled many typical *Enterobacter* spp. (decreased susceptibility to ampicillin, cefazolin, amoxicillin-clavulanic acid, and trimethoprim-sulfamethoxazole) and correlated with successful treatment regimens of levofloxacin or meropenem. Both isolates exhibited decreased susceptibility to colistin and demonstrated variable susceptibility to third-generation cephem agents such as ceftazidime and cefotaxime.

The initial description of *Enterobacter intestinihominis* sp. nov. (50) was published in spring of 2025 but did not have corresponding phenotypic (including Gram reaction or cultivation conditions) or clinical data. The taxon was derived from human gut microbiome studies. In early 2026, Cui et al. (59) reported isolation of two strains of *E. intestinihominis* from urine of a unique intensive care unit patient in China who was undergoing ceftazidime therapy. Both isolates had elevated minimum inhibitory concentration (MIC) values for piperacillin-tazobactam, ceftriaxone, cefepime, aztreonam, amikacin, tobramycin, ciprofloxacin, levofloxacin, trimethoprim-sulfamethoxazole, and ceftazidime. Highly variable MIC values were noted between the two isolates for meropenem and imipenem, manifested by differential possession of *bla*_KPC-2_ and *bla*_KPC-33_ determinants and discordant results by two commercial carbapenemase rapid immunochromatographic systems. Ceftazidime selective pressure was not only implicated in the generation of one *E. intestinihominis* strain that was resistant to ceftazidime-avibactam but also in the transmission of IncN/FII plasmids within *E. intestinihominis* that harbor *bla*_KPC-2_/*bla*_KPC-33_.

A United States reference laboratory added three species to the family *Enterobacteriaceae* genus *Dryocola* (153, 154). While antecedent clinical data were not available, the three novel isolates (27) were derived from knee specimens of two separate patients and a foot specimen from a third. Initial MALDI-TOF MS characterization of the three isolates yielded a variety of *Buttiauxella* spp. with scores less than 1.82. Partial 16S rRNA gene PCR sequencing results suggested organisms such as *Pantoea agglomerans*, *Citrobacter koseri*, and even *Salmonella enterica*. Whole genome sequencing of the three isolates indicated optimal clustering with the aforementioned *Dryocola* genus and less similarity with *Cedecea* spp. The three novel species tolerated 10% sodium chloride and exhibited variable Voges-Proskauer and arginine dihydrolase reactivity. One of the novel taxa, *Dryocola mayonis* sp. nov., was coined relative to the reference laboratory, while the other two taxa, *Dryocola baronae* sp. nov. (155) and *Dryocola sharpae* sp. nov. (156), are perhaps responses to a recent publication (157) that highlighted a relative scarcity of taxonomic designations that reflect the accomplishments of female scientists. The three novel *Dryocola* spp. taxa were broadly susceptible to antimicrobial agents, with variability noted with cefazolin and ampicillin.

Of four non-glucose fermentative Gram-negative bacilli that were newly included in 2025, only *Stenotrophomonas muris* sp. nov. was shown to have clinical relevance. However, this determination was only offered by way of follow-up literature (70), as the initial description of this taxon by Afrizal et al. in 2022 (69) was derived from a gnotobiotic murine model and offered no phenotypic description. Two Gram-negative bacillus isolates, previously identified as *Stenotrophomonas maltophilia* by both commercial biochemical profiling and MALDI-TOF MS, were recovered from blood culture and sputum culture in two individual patients (70). The former isolate came in the context of a third-decade male diabetic patient with diagnoses of septic shock, severe pneumonia, disseminated mucoromycosis, and multiple organ failure, which emanated from an initial skin ulceration that eventually required amputation. The second isolate was derived from a patient with a seven-month history of acute lymphoblastic anemia. Upon admission, multiple hepatic cysts were noted on imaging studies, and examination revealed clear lung sounds. The patient was treated with chemotherapy during the hospitalization but developed myelosuppression and lung infection. The identity of the two isolates was later determined to be *S. muris* by whole genome sequencing. Both isolates exhibited *in vitro* susceptibility to minocycline and ceftazidime (per CLSI M100 Other Non-*Enterobacterales* breakpoints), while each generated an MIC of 8 μg/mL for trimethoprim-sulfamethoxazole (resistant). The bloodstream isolate yielded an intermediate interpretation from levofloxacin broth microdilution testing. The researchers utilized EUCAST *Pseudomonas* spp. guidelines to report resistance interpretations for colistin and polymyxin B susceptibility testing. Of further interest, a variety of *in vitro* virulence assays, as well as a murine model, were challenged by the two clinical isolates and an ATCC strain of *S. maltophilia*. The two *S*. *muris* isolates demonstrated increased virulence capacity over that of *S. maltophilia*, with the bloodstream isolate showing significantly enhanced pathogenicity versus the respiratory isolate.

The fusiform Gram-negative bacillus *Capnocytophaga endodontalis* sp. nov. (57). was isolated by practitioners in the Republic of Korea from a periapical abscess in the left mandibular second molar area. The isolate was cultivated on tryptic soy agar containing several supplements in an anaerobic chamber. Despite *Capnocytophaga* spp. being recognized as facultative organisms (158), no attempts were made to subculture this novel isolate in aerobic conditions. No data regarding potential isolation of additional pathogens, antimicrobial management, or clinical outcome were provided.

A total of 74 novel prokaryotes are listed in Tables 3 and 5 for which obligate anaerobic status was documented. Of these, 44 and 27 are Gram-positive and Gram-negative, respectively, with three obligate anaerobes not having a published Gram reaction (Table 5). Very commonly, these microbes were isolated from fecal specimens in the context of microbiome studies. Up to eight taxa recovered in routine clinical practice will be discussed in the following paragraphs.

A report from the Netherlands (72) described four novel *Anaerococcus* spp. from a variety of clinical specimens (Table 3). Blood culturing recovered *Anaerococcus cruorum* sp. nov. from three patients with respective findings of prosthetic aortic valve endocarditis, fever, and pneumonia (underlying MALT lymphoma). In the patients with fever and pneumonia, *A. cruorum* was isolated from 50% of collected blood culture sets and was viewed by providers as being non-significant. In the case of endocarditis, this Gram-positive coccus was isolated in 1 of 16 blood culture sets, while the implicated agent of disease, *Propionibacterium avidum*, was recovered in 11 of 16 blood culture sets. In contrast, *Anaerococcus groningensis* sp. nov. was detected in all collected blood culture sets from two patients. The bacterium was considered non-significant in both a patient with SARS-CoV-2 infection and in a second patient with co-morbidities that included pulmonary embolism, ischemic cardiovascular accident, ascites, and suspected metastatic pancreatic carcinoma. *Anaerococcus kampingiae* sp. nov. was isolated from culture of sphenoid sinus purulent material from a male patient and from culture of abscess material near a prosthetic hip joint. In both cases, several additional organisms were cultivated, placing skepticism on the relevance of *A. kampingiae*. Moreover, post-culture diagnosis of the sphenoid sinus work-up was sinusitis due to *Serratia marcescens*. Finally, three isolates of *Anaerococcus martiniensis* sp. nov. were recovered from cancer patients (one patient with renal cell carcinoma and lung carcinoma; one patient with esophageal carcinoma) and a febrile patient with epilepsy. Despite being the only organism isolated from ascites fluid and from one of two blood culture sets, *A. martiniensis* relevance in the esophageal carcinoma patient and the patient with epilepsy, respectively, was deemed non-significant. Isolation of this obligate anaerobe from thoracal debris was concomitant with two other bacterial species and *Aspergillus fumigatus*. Clinical relevance was ascribed to the latter three microbes.

*Finegoldia dalianensis* sp. nov. (81). was isolated from a purulent skin abscess in a hospitalized female patient. The isolate was initially identified as *Finegoldia magna* via MALDI-TOF MS. A 99.7% 16S rRNA gene sequence similarity with a *F. magna* type strain was observed; however, less than 91% ANI value was determined and a number of biochemical differences were noted. Subsequent phylogenetic comparison of *F. dalianensis* with 82 archived *F. magna* constructs indicated that several of the *F. magna* isolates actually aligned more closely with the new taxon. From a patient with oral mucositis receiving high-dose cytarabine chemotherapy for acute myeloid leukemia, blood cultures were collected during fever spikes. A single blood culture cultivated the spore-forming, anaerobic Gram-positive bacillus that was identified, at the time, as *Lachnoanaerobaculum gingivalis* (159). The organism easily decolorizes and may appear Gram-negative. Illness resolved with meropenem therapy. A difference of 11 base pairs in the second half of the 16S rRNA gene sequence when compared with a type strain prompted phylogenetic analysis. ANI values less than 94% when compared with other *Lachnoanaerobaculum* spp. resulted in the novel designation *Lachnoanaerobaculum sanguinis* sp. nov. (83).

*Terrisporobacter muris* sp. nov. is a second taxon originally reported from murine gnotobiotic studies of Afrizal et al. (69) that has newfound relevance in human clinical specimens. This anaerobic Gram-variable bacillus was reported in a case of sepsis from a patient with a sigmoid colon perforation and history of lung adenocarcinoma with chemotherapy intervention (91). Two sets of blood cultures yielded tiny, gray colonies upon blood agar and Brucella agar subculture. A MALDI-TOF MS identification of *Clostridium glycolicum* (a former designation of *Terrisporobacter glycolicus*) was considered inconclusive due to a limited library. Whole genome sequencing produced a definitive identification of *T. muris*. Susceptibility testing performed by an epsilometric method yielded MIC values of >32 μg/mL for ceftriaxone, 24 μg/mL for cefoxitin, >32 μg/mL for trimethoprim-sulfamethoxazole, 6 μg/mL for meropenem, 0.064 μg/mL for metronidazole, 0.25 μg/mL for vancomycin, and 1 μg/mL for penicillin.

The obligate anaerobic Gram-negative bacillus *Dentiradicibacter hellwigii* gen. nov., sp. nov. (96). was published by a collaboration of dental professionals from Germany and Switzerland. A female patient was noted to have two teeth with secondary endodontic infections. Root canal contents were disinfected and specimens were collected with sterile paper points. *D. hellwigii* was isolated from the root canal of the upper left maxillary premolar. Growth of the organism was noted to be poor on media such as Columbia blood agar or fastidious anaerobe agar with 7% defibrinated horse blood, but was significantly improved through co-culture with *Capnocytophaga sputigena*, *Capnocytophaga granulosa*, or *Prevotella intermedia*.

The obligate anaerobic Gram-negative genera *Desulfovibrio* (160), *Porphyromonas* (161), and *Ruthenibacterium* (162) have been shown to be present in increased numbers in patients with Parkinson’s disease. Moreover, investigations centering around the nematode *Caenorhabditis elegans* revealed that *Desulfovibrio* spp. isolated from individuals with Parkinson’s disease enhanced aggregation of α-synuclein fibrils (a key pathologic feature of Parkinson’s disease) more so than *Desulfovibrio* spp. derived from healthy individuals (163). *Porphyromonas gingivalis* has been implicated in the hypercoagulation and systemic inflammation found in Parkinson’s disease (164, 165). Novel *Desulfovibrio* spp. and *Porphyromonas* spp. taxa, along with *Mediterraneibacter flintii* sp. nov. and the novel genus-level taxon *Owariibacterium komagatae* gen. nov., sp. nov., were characterized by Hamaguchi et al. (97) from feces of a 76-year-old patient with Parkinson’s disease. Mechanistic studies were not undertaken in this publication; however, when compared with persons without Parkinson’s disease, the relative abundance of individual *Desulfovibrio falkowii* sp. nov., *M. flintii*, and *O. komagatae* in fecal matter was increased (*P* ≤ 0.0073).

Both bacillus and spiral forms of the Gram-negative pathogen *Fluviispira vulneris* sp. nov. (Table 4) have been observed on microscopy. This taxon was first published in 2023 (104) and included in 2025 (71). An elderly man presented to a Chinese hospital for right calf pain and bleeding. Systemic inflammatory markers that were increased included procalcitonin, leukocyte count (left shift), and C-reactive protein. Necrotic tissue debridement generated this aerobic, red-pigmented isolate. The isolate was reported to be resistant to aztreonam and gentamicin, while susceptible to ampicillin, ceftriaxone, ciprofloxacin, tetracycline, and imipenem when using a testing method not directly advocated by CLSI.

### Taxonomic revisions

Traditionally, the *Streptococcus mitis* group complex has contained eight species, namely *Streptococcus mitis*, *Streptococcus oralis*, *S. pneumoniae*, and *S. pseudopneumoniae*, in addition to the more distant *Streptococcus infantis*, *Streptococcus peroris*, *Streptococcus australis,* and *S. parasanguinis*. As noted within this manuscript (Table 1) and prior publications, based mostly upon the study of single isolates, newer additions to this group have included *Streptococcus toyakuensis*, *S. thalassemiae*, *Streptococcus chosunensis*, *Streptococcus gwangjuensis*, *S. humanilactis, Streptococcus bouchesdurhonensis*, and *S. vulneris* (40). Kilian and others have noted the genetic diversity within *S. mitis*, *S. oralis,* and *S. infantis* using traditional taxonomic standards (DNA-DNA homology and ANI/dDDH boundaries), which is somewhat unique to this group of organisms. Kilian and colleagues revisited the classification of *S. mitis* and the recently described related species (40). The authors performed whole genome sequencing and phylogenetic analysis of aligned core genomes of an unidentified clinical isolate compared with *S. mitis, S. pneumoniae*, and related species in the *S. mitis* complex. They compared the isolate’s genome sequence to 221 genome sequences of the *S. mitis* complex in the GenBank database, selecting only high-quality sequences. In addition, they mapped selective genes specific for *S. pneumoniae* and subgroups of *S. mitis*. After removing redundant sequences, 100 high-quality genome sequences were selected for more detailed analysis (40). The authors identified 11 clusters (labeled Ia, Ib, IIa, IIb, IIc, and III, in addition to new clusters labeled IV, V, VI, VII, VIII). As mentioned above, the authors noted complex diversity for the *S. mitis* group clusters with ANI and dDDH values that do not allow for distinct separation into species. They proposed amending the rules for the *S. mitis* complex (lowering the values for ANI and dDDH) to allow for the observed genetic diversity and clonal evolution within clusters. As such, the previously named *S. chosunensis*, *S. toyakuensis*, *S. gwangjuensis*, *S. humanilactis*, and *Streptococcus hohhotensis* are now considered heterotypic synonyms of *S. mitis* (Table 6). The newly described *S. nakanonensis* and *S. parapneumoniae* (Table 1) are heterotypic synonyms of *S. thalassemiae*. Finally, *Streptococcus downii* is a heterotypic synonym of a subspecies of *S. oralis*, namely *S. oralis* subsp. *dentisani*. The authors provide a detailed emended description of *S. mitis* (40).

Three Gram-positive bacilli have also been reassigned to existing taxa. *Pseudonocardia* spp. are primarily recovered from soil and plant ecosystems (113). *Pseudonocardia carboxydivorans,* now considered a subspecies of *Pseudonocardia alni* (115), has been associated with meningitis complicating a traumatic brain injury in a 67-year-old man who cultivated vegetables. The cerebrospinal fluid analysis was suggestive of acute inflammation, and the organism grew after 7 days of incubation. Several weeks later, upon repeat lumbar puncture, the organism was cultivated again, confirming its role as the causative agent of this patient’s meningitis. The patient improved after 10 months of trimethoprim/sulfamethoxazole treatment (114). No other human infections associated with *P. alni* subsp. *carboxydivorans* comb. nov. have been reported.

*Virginibacillus massiliensis,* originally isolated from feces, has been reclassified as a heterotypic synonym of *Virigibacillus salexigens* (118). There are no reports of this organism causing infections in humans. Six strains of actinobacteria recovered from various sources including wounds, urine, and human blood were determined to form a phylogenetically separate branch in the family *Microbacteriaceae* (119). The details of the clinical presentations were not provided. The novel isolates were assigned to a new genus *Zimmermannella* and four new species *Z. alba*, *Z. bifida*, *Z. helvola* and *Z. faecalis* were designated (119). However, in a later study by Vandamme (120), the authors determined by phylogenetic, proteomic, and phenotypic analyses that *Z. alba* represents a *Pseudoclavibacter* species, and they proposed its reclassification as *Pseudoclavibacter albus* comb. nov.

The Gram-negative bacterium *Roseomonas aerofrigidensis* was originally characterized following isolation from an air conditioner in South Korea (121). This organism is mentioned in this discussion because of a recent case report (122) that documented its isolation in infectious endocarditis in Brazil. Liu and Xin (21) proposed the designation *Teichococcus aerofrigidensis* comb. nov. for this taxon. Interestingly, in the phylogenetic analysis performed by Sacramento et al. (122), the endocarditis isolate displayed a higher degree of similarity to several species of the *Teichococcus* genus than to *Roseomonas* spp.

With respect to anaerobic bacteria, initial isolation and characterization of the anaerobic Gram-negative coccus *Megasphaera jansseni* sp. nov. from the wild boar *Sus scrofa* (74) prompted taxonomic investigation of other members of families *Veillonellaceae* and *Sporomusaceae*. This resulted, in part, in proposals of *Megasphaera geminata* comb. nov., *Dialister histaminiformans* comb. nov., and *Anaerosinus gibii* comb. nov.

### Recently-ascribed and additional clinical significance

*Faucicola mancuniensis* was initially derived from tonsils from a healthy subject, with valid and effective publication in 2015 (166), while the aerobic Gram-positive bacillus *Gulosibacter massiliensis* was effectively published by Yacouba et al. (167) in 2023 and included in 2024 (168). Updates to these two organisms (Table 7) include *F. mancuniensis* being isolated from a pharyngeal swab of a healthy individual in the context of meningitis surveillance of close contacts and surrounding populations (141). Susceptibility testing of this Gram-negative coccus generated MIC values ≤ 1 μg/mL for 25 of the antimicrobial agents tested. Li et al. (147) reported on an emergent instance of a man stepping into a gutter, developing pain in the right foot with obvious bleeding. Innate biomarkers were not elevated at the time of presentation. An operative procedure for hemostasis, infection avoidance, and toe flap trimming noted that no fracture was present and that a web space wound had reached the bone surface. However, exudate from the wound increased post-operatively, resulting in culture and isolation of *G. massiliensis*. Empiric cefuroxime and metronidazole were transitioned to oral levofloxacin (0.5 g qd) upon release of culture results that were augmented by 16S rRNA gene sequencing of the isolate.

Three additional publications make note of emerging antimicrobial resistance determinants among Gram-negative bacilli. Both *E. chengduensis* (169, 170) and *Enterobacter chuandaensis* (171) were effectively published and included in 2019. With respect to the former, Fu et al. (142) characterized four additional *E. chengduensis* isolates (all previously reported by clinical laboratories as *Enterobacter cloacae*) derived from blood, urinary tract, intraperitoneal, and indwelling catheter specimens. One of the patients expired during intensive care unit residence. The four isolates were resistant to imipenem and meropenem (MIC > 8 μg/mL), as well as ertapenem (MIC > 4 μg/mL), but susceptible to aztreonam. All isolates carried a *bla*_NDM-1_ genotype. Intrahospital clonal transmission was suggested by single nucleotide polymorphism analysis. Chen and colleagues (143) described a single isolate of *E. chuandaensis* derived from a perianal swab that exhibited resistance to all tested β-lactam agents (including meropenem, imipenem, aztreonam, ceftazidime-avibactam), fluoroquinolones, tetracycline, and trimethoprim-sulfamethoxazole. *In vitro* susceptibility was noted for colistin, tigecycline, and amikacin. The authors recognized the combination genotype of *bla*_KPC-2_ and *bla*_NDM-1_ as being novel among carbapenem-resistant *Enterobacter cloacae* complex organisms. The taxon *Pseudomonas juntendi* was first reported from clinical specimens in the Eastern Hemisphere in 2019 (172). Several subsequent reports have noted multiple means of β-lactam (including carbapenem) resistance within clinical isolates worldwide (173–178). A recent publication (144) describes a urinary *P. juntendi* isolate (susceptible only to tigecycline, minocycline, and polymyxin B) with phylogenetic characterization consistent with an East Asian lineage and potential regional dissemination. *bla*_IMP-15_ and *bla*_NDM-1_ determinants were localized on a single chromosome. The authors postulated *P. juntendi* as an emerging reservoir for metallo-β-lactamases due to its resistance island capacity and phenotypic traits such as biofilm formation and bile salt tolerance.

*Pandoraea pneumonica* was initially derived from a cystic fibrosis patient, effectively published by Peeters et al. (179) in 2019, and included in 2020 (180). Extensive clinical data, however, were not provided. A publication in 2025 (145) summarizes a 2021-2024 outbreak of *P. pneumonica* bloodstream infection in China involving 30 patients. The authors noted that over 70% of patients were afflicted during flood season (May 15 to November 15), a time significant for outbreaks of infectious diseases within vulnerable communities, serving as a potential portal of entry for *P. pneumonica*. The same proportion of patients were either male or ≥50 years of age. Two-thirds of patients were farmers, while one half of patients were previously diagnosed with hypertension. The majority of patients had concomitant pulmonary infection. In this case series, five patients expired, though co-infection with SARS-CoV-2 and *Burkholderia cepacia* was documented in one of the pulmonary cases. Presented MIC_50_ data for 28 isolates, presumably generated by broth microdilution testing, included ≥128 μg/mL for ceftazidime, ≥16 μg/mL for meropenem, 2 μg/mL for imipenem, 4 μg/mL for ciprofloxacin, 8/4 μg/mL for piperacillin-tazobactam, and ≥16 μg/mL for gentamicin.

The *Corynebacterium hesseae* taxon had its origin largely in mucocutaneous specimen sources (181). Araújo et al. (146) chronicled a febrile elderly male patient in Brazil who presented for emergent care with tachycardia and tachypnea. Microbial growth was detected in two sets of blood culture bottles (two total sets collected) after 22 h of incubation. MALDI-TOF MS identified subcultured colonial growth as *Corynebacterium aurimucosum*. Subsequent ANI and DNA-DNA hybridization values relative to a *C. hesseae* type strain were 96.36% and 84.9%, respectively. Phylogenetic analysis clustered the clinical isolate among six deposited *C. hesseae* genomes from the United States and Europe and apart from that of *C. aurimucosum*. No other organisms were isolated from blood cultures. Disk diffusion antimicrobial susceptibility testing was rendered per guidelines from the Brazilian Committee for Antimicrobial Susceptibility Testing. Resistance was reported for penicillin, tetracycline, clindamycin, and ciprofloxacin. Susceptibility was reported for rifampin, vancomycin, and linezolid. Biofilm production was ascertained through *in vitro* experimentation, while challenge inocula of > 10^9^ cells caused rates of mortality exceeding 30% in a *Galleria mellonella* larva model system.

## CONCLUSION

The *Journal of Clinical Microbiology* remains committed to an annual review of salient findings pertinent to both proposed taxonomic revisions and novel organism elucidation. Consistent with previous iterations of this compendium, many novel taxa have been characterized from human microbiome research and their roles in health and/or disease states require future study. As highlighted throughout the document, a concerning observation among some of the novel taxa is the acquisition of multidrug resistance. Comprehensive phylogenetic analyses have noted that species previously characterized as “novel” are, in fact, synonyms of existing species. While the College of American Pathologists has recently amended checklist requirements relative to microbial nomenclature (182), consistency therein remains important for commercial identification systems, interpretation of antimicrobial susceptibility testing data, and public health notification.
